# The Role of Ceramides in Metabolic and Cardiovascular Diseases

**DOI:** 10.3390/jcdd13010030

**Published:** 2026-01-04

**Authors:** Manuel Gonzalez-Plascencia, Idalia Garza-Veloz, Virginia Flores-Morales, Margarita L. Martinez-Fierro

**Affiliations:** Molecular Medicine Laboratory, Academic Unit of Human Medicine and Health Sciences, Universidad Autónoma de Zacatecas, Carretera Zacatecas-Guadalajara Km.6. Ejido la Escondida, Zacatecas 98160, Mexico; manuelgonzalezcharro@gmail.com (M.G.-P.); virginia.flores@uaz.edu.mx (V.F.-M.)

**Keywords:** ceramides, sphingolipids, cardiovascular risk, cardiovascular disease, metabolism, biomarkers

## Abstract

Ceramides are bioactive sphingolipids increasingly recognized as mediators of cardiometabolic disease and residual cardiovascular risk. Accumulating evidence from experimental and clinical studies indicates that specific ceramide species contribute to insulin resistance, endothelial dysfunction, myocardial injury, and adverse cardiovascular outcomes. In particular, long-chain ceramides (C16:0, C18:0, C20:0 Cer) are consistently associated with myocardial infarction, heart failure, and cardiovascular mortality, whereas very-long-chain ceramides (C22:0, C24:0 Cer) exhibit neutral or potentially protective associations. This narrative review integrates biochemical, experimental, and clinical evidence to examine ceramide metabolism, molecular diversity, and their emerging role as biomarkers for cardiovascular risk stratification. We also discuss ceramide-based risk scores and their potential clinical utility beyond traditional lipid parameters. Understanding the structure–function relationships of ceramides may support the development of novel diagnostic and therapeutic strategies in cardiovascular prevention.

## 1. Introduction

Ceramides (Cer), bioactive lipids from the sphingolipid family [1], have recently attracted growing attention in the cardiovascular field [2]. They have two main functions: first, they serve as structural precursors of complex sphingolipids [3]; second, they act as regulatory molecules, sending signals inside the cell that affect processes such as the cell cycle, programmed cell death (apoptosis), and fatty acid metabolism [3,4]. When nutritional overload or cellular stress occurs, ceramide production rises above normal levels and becomes harmful to cells [1,5,6]. Roger Unger, who coined the term lipotoxicity, described ceramide as “the most damaging lipid” because of its requirement for lipid-induced apoptosis [7].

Cer accumulation contributes to pathological processes with important clinical implications. At the systemic level, it promotes atherosclerosis and chronic inflammation [5,8]. At the metabolic level, it promotes insulin resistance [9]. At the cellular level, it disrupts the cell cycle and activates apoptosis [1,10]. These mechanisms are linked to the development of metabolic and cardiovascular diseases (CVD), including obesity, type 2 diabetes mellitus (T2DM), metabolic dysfunction–associated steatotic liver disease (MASLD), stroke, atherosclerotic cardiovascular disease (ASCVD), chronic kidney disease (CKD), coronary artery disease (CAD), myocardial infarction (MI), and heart failure (HF) [9,11,12,13,14].

According to the 2023 Global Burden of Disease study, cardiovascular-related deaths worldwide reached 19.2 million in 2023 [15]. In this context, long-chain ceramides (LCCs) (for example, C16:0, C18:0, C20:0 Cer) are associated with higher risk of major adverse cardiovascular events (MACE), while very-long-chain ceramides (VLCCs) (such C22:0, C24:0 Cer) appear to exert more protective effects [16,17,18,19,20]. This functional diversity has driven the search for new molecular biomarkers to refine risk stratification and strengthen cardiovascular preventive strategies.

## 2. Methods

This review sought to clarify the biological functions of ceramides and synthesize experimental, clinical, and epidemiological evidence on their role in metabolic and cardiovascular disease, with emphasis on their potential as biomarkers and therapeutic targets. A comprehensive search was conducted in PubMed and Scopus without time restrictions using MeSH terms and free-text keywords (“ceramides,” “sphingolipids,” “cardiovascular disease,” “cardiovascular risk,” and “metabolism”).

The selection of the studies was based on conceptual relevance, originality, and clinical or mechanistic contribution. Inclusion criteria comprised the following: (1) reviews or experimental studies that elucidate the metabolism or function of ceramides; (2) observational or interventional clinical studies that evaluate ceramide in metabolic or cardiovascular outcomes; and (3) studies with epidemiological data that report associations with cardiovascular events or mortality. Exclusion criteria included non-peer-reviewed sources, duplicate datasets, studies lacking sufficient methodological detail, and studies where ceramides were not a primary focus. Although a formal PRISMA workflow was not applied due to the narrative design, a qualitative appraisal was performed prioritizing studies with adequate sample size, validated lipidomic methodologies, and clearly defined outcomes. The limitations inherent to this narrative approach are acknowledged.

## 3. Biochemical and Physiological Bases

### 3.1. Background

Sphingolipids are a class of lipids present in the plasma membrane of eukaryotic cells. They are among the most abundant membrane lipids, together with glycerophospholipids and cholesterol [21]. Major members include sphingomyelin and glycosphingolipids (sugar-linked sphingolipids), such as cerebrosides and gangliosides [22,23]. In 1884, Johan L.W. Thudichum identified various lipid compounds in the human brain, one of which he named sphingosine, inspired by the enigmatic nature of the Sphinx, the mythical Greek creature [24]. Its precise chemical structure was not clarified until 1947, when Herb Carter characterized it in detail [24]. These discoveries laid the foundation for modern sphingolipid biology.

Sphingosine has an amphipathic structure, with a polar head and a hydrophobic chain. This backbone forms sphingoid bases, the precursors of the sphingolipid family [6,23]. Ceramide, the central building block of sphingolipids, is synthesized in the endoplasmic reticulum (ER) and functions as a second messenger in multiple signaling pathways [25]. Its involvement in processes such as differentiation, proliferation, and apoptosis has been demonstrated in various studies [26,27]. The recognition of these functions took nearly a century, until the 1980s and 1990s, when the work of Hannun, Obeid, and Kolesnick established ceramide’s role in cell signaling [4].

### 3.2. Chemical Structure of Ceramides

**Figure 1:**
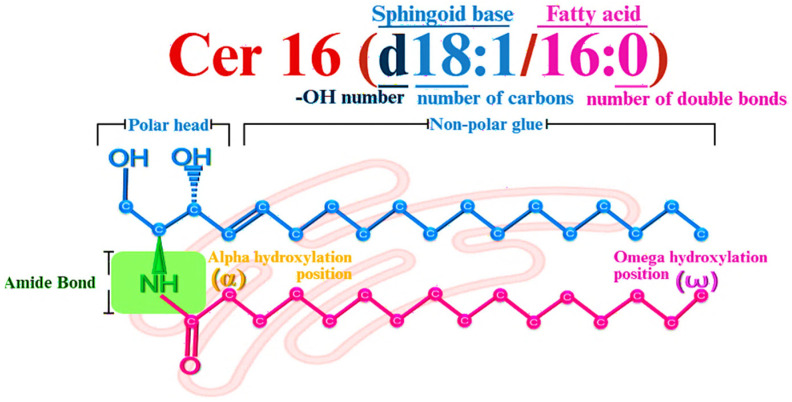
Nomenclature and molecular structure of ceramide 16 (Cer 16; d18:1/16:0). The molecule consists of two main components: the sphingoid base (blue), an 18-carbon chain with a trans Δ4 double bond (d18:1), and the N-acyl chain (pink), a saturated 16-carbon fatty acid (16:0).The polar hydroxyl groups at C1 and C3, the amino group at C2, and the amide bond (−NH−CO−) connecting the sphingoid base with the fatty acid are indicated. Also shown are the α (C2) and ω (terminal carbon) hydroxylation positions, which generate structurally relevant biological variants.

Ceramides display considerable functional versatility despite their relatively simple chemical architecture, which allows for extensive molecular diversity [28]. At their core, ceramides are formed by the covalent linkage of a sphingoid base—a long-chain amino alcohol—to a fatty acid through a stable amide bond [29,30], as illustrated in Figure 1.

This N-acylation reaction is catalyzed by ceramide synthase enzymes and can be reversed by ceramidases, enabling the dynamic regulation of ceramide levels [31,32]. Through these reactions, ceramides serve as central metabolic intermediates in the synthesis and degradation of complex sphingolipids, including sphingomyelins, glucosylceramides, and sphingosine 1-phosphate [24,33].

The combination of a polar head group and two hydrophobic chains gives ceramides an amphipathic character. Their small polar head contains hydroxyl groups and an amino group at the C2 position, while the hydrophobic region is formed by the sphingoid base and the fatty acid chains [29]. This molecular organization produces a conical shape, which facilitates insertion into lipid bilayers and influences membrane curvature and lipid microdomains organization [10,23,31].

Structural diversity in ceramides arises primarily from variations in the sphingoid base and the N-acyl chain. Most sphingoid bases contain 18 carbon atoms and form the backbone of all sphingolipids [23,34]. Sphingosine (Sph), the predominant sphingoid base in mammals, contains a Δ4-trans double bond that increases molecular rigidity and affects lipid packing [28]. In contrast, sphinganine or dihydrosphingosine (dhSph) lacks this double bond, resulting in the greater flexibility and reduced bioactivity of dihydroceramides (dhCer) [24]. Additional variants, such as phytosphingosine and 6-hydroxysphingosine, further expand structural diversity, particularly in the epidermis, where they contribute to skin barrier function [29,35].

Further variability is introduced by the N-acyl chain, which can range from 14 to 36 carbon atoms. Very long and ultra-long chains are especially prevalent in human epidermal ceramides [10]. The degree of saturation of these chains influences membrane organization, with saturated chains promoting rigid lipid domains and unsaturated chains increasing membrane fluidity [31,36]. Bases on the presence and position of hydroxyl groups, ceramides are classified as non-hydroxylated, α-hydroxylated, or ω-hydroxylated [29,37]. Together, these structural variations determine the unique biophysical properties of ceramides, which are essential for both for their metabolic roles and their structural function in maintaining membrane stability [24].

### 3.3. Nomenclature and Classification of Ceramides

The nomenclature of ceramides follows the recommendations of the International Union of Pure and Applied Chemistry (IUPAC) and the International Union of Biochemistry and Molecular Biology (IUBMB), under the supervision of the Joint Commission on Biochemical Nomenclature (JCBN), which is responsible for the standardized naming of biochemical compounds [38,39]. Within this framework, the LIPID MAPS classification system organizes lipids into eight major categories and places ceramides as a core subclass of sphingolipids, from which numerous species are derived [39,40].

To simplify structural description, sphingoid bases are represented using an abbreviated notation that summarizes their key features. The prefix ‘d’ denotes1,3-dihydroxylated structures, such as Sph and dhSph [24,41], whereas the prefix ‘t’ corresponds to 1,3,4-trihydroxylated bases, such as phytosphingosine [39,40]. Carbon chain length and the number of double bonds are expressed as (*n:x*), where *n* indicates the number of carbons and *x* the number of unsaturations [41]. Accordingly, sphingosine is designated as d18:1, dihydrosphingosine as d18:0, and phytosphingosine as t18:0 [37,42]. By convention, this notation assumes the natural D-erythro (*2S*,*3R*) stereochemistry, while non-canonical stereoisomers require full systematic naming [24,41].

Additional structural diversity arises from the N-acyl chain. Ceramides are classified as non-hydroxylated (N), *α*-hydroxylated (A), or *ω*-hydroxylated (O), depending on the presence and position of hydroxyl groups within the fatty acid moiety [29,36]. Fatty acid chains are described using the format C(*n*):*x*, where *n* indicates chain length and *x* the number of double bonds. For example, palmitic acid is denoted as C16:0 and oleic acid as C18:1 [24,39]. The LIPID MAPS system integrates both structural components into a unified notation, expressed as Cer(base/acyl). Thus, a ceramide composed of Sph and palmitic acid is represented as Cer(d18:1/16:0), enabling precise identification of molecular species [39,41].

Accurate nomenclatural is essential not only for classification but also for linking specific ceramide species to distinct biological functions. However, the classification of ceramides based on N-acyl chain length lacks universal consensus and varies across research fields. In general biochemistry, fatty acids are classified as short-chain (C2:0–C6:0), medium-chain (C6:0–C12:0), or long-chain (≥C13:0) [43]. In contrast, studies in metabolic pathophysiology commonly distinguish medium-chain (C12:0–C14:0 Cer), long-chain (C16:0–C18:0 Cer), very-long-chain (C20:0–C24:0 Cer), and ultra-long-chain (≥C26:0 Cer) ceramides.

Within this metabolic context, ceramides containing the C16:0 and C18:0 acyl chains have been associated with lipotoxicity, whereas the very-long-chain species (C22:0–C24:0) display distinct effects on mitochondrial function, oxidative stress (OS), and apoptosis [4,8,44]. In dermatology, classification shifts toward even longer chains, as the integrity of the epidermal barrier depends on ceramides with ultra-long-chain ceramides (ULCCs), typically ≥C26:0 and extending up to 34 to 36 carbons [29,45]. Consequently, ceramides considered “long-chain” in metabolic studies are functionally short in the context of skin biology, where ultra-long chains are required and whose presence manifests in lamellar lipid organization in the stratum corneum (SC) [29,45].

### 3.4. Synthesis

**Figure 2:**
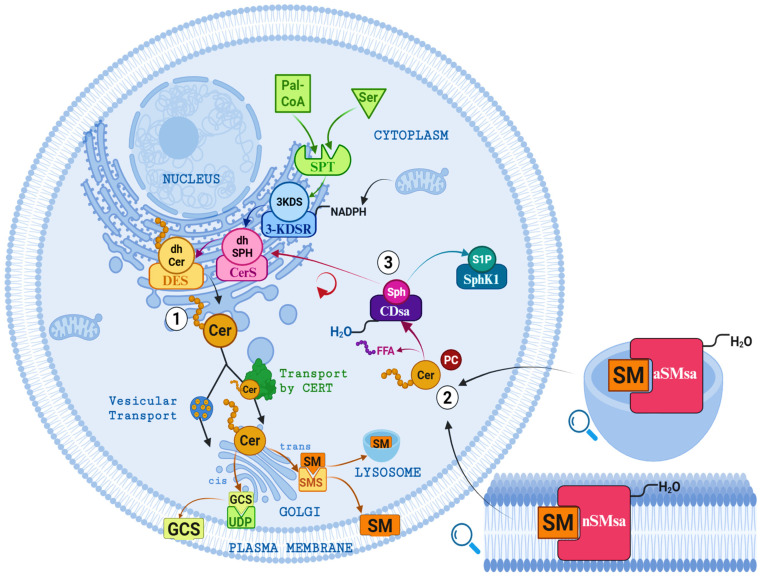
Ceramide (Cer) synthesis in the eukaryotic cell occurs via three main pathways: (1) de novo synthesis in the ER, where serine (Ser) and palmitoyl-CoA (Pal-CoA) condense via serine palmitoyltransferase (SPT) to form 3-ketosphinganine (3-KDS), which is then reduced by 3-ketosphinganine reductase (3-KDSR), which is reduced by 3-KDSR to dihydrosphingosine (dhSph). Ceramide synthase (CerS) then acylates dhSph to produce dihydroceramide (dhCer), which is desaturated by DES to yield Cer. (2) Sphingomyelin hydrolysis, mediated by neutral or alkaline sphingomyelinase (SMase), degrades sphingomyelin (SM) in lysosomes or the plasma membrane, releasing Cer and phosphocholine. (3) Salvage pathway, where lysosomal breakdown of glycosphingolipids and SM generates sphingosine, which is either re-acylated by CerS to regenerate Cer or phosphorylated by sphingosine kinase (SphK1) to form sphingosine-1-phosphate (S1P).

Ceramide synthesis does rely on a single biochemical route. Instead, it occurs through an interconnected metabolic network that ensures adequate availability under both physiological and pathological conditions [10]. This network includes three functionally linked pathways: de novo synthesis from serine and palmitoyl-CoA, sphingomyelin (SM) hydrolysis mediated by sphingomyelinases, and the salvage pathway, which recycles sphingosine [24,26,30]. An overview of these pathways is provided in Figure 2.

#### 3.4.1. De Novo Pathway

This represents the principal anabolic route for ceramide synthesis from simple precursors. It is conserved across eukaryotic cells and is essential for maintaining the basal pool of structural sphingolipids [46]. This pathway occurs on the cytosolic side of the ER and proceeds through four sequential enzymatic steps [2,6]:

- Condensation: the initial and rate-limiting step is catalyzed by serine palmitoyltransferase (SPT), a multi-protein enzyme complex regulated by the SPTLC1–3 subunits and the small proteins ssSPTa/b. Under physiological conditions, SPT preferentially uses palmitoyl-CoA (C16:0) and serine as substrates. However, the presence of SPTLC3 allows for the utilization of alternative fatty acyl-CoAs or amino acids, leading to the formation of atypical sphingolipid species, including 1-deoxysphingolipids [6,21,47]. The reaction produces 3-ketosphinganine (3-KDS) and establishes a direct link between saturated fatty acid availability and ceramide biosynthesis [48].
- Reduction: 3-Ketosphinganine is rapidly reduced to sphinganine (dihydrosphingosine) by the NADPH-dependent enzyme 3-ketosphinganine reductase (3-KDSR). This step generates the first stable sphingoid base of the pathway [3,10,24].
- N-acylation: Subsequently, ceramide synthases (CerS1–6) catalyze the attachment of fatty acyl chains of defined lengths, ranging from 14 to 34 carbons atoms, generating dihydroceramides (dhCer). Each CerS isoform displays substrate specificity and tissue-dependent expression, thereby shaping the characteristic ceramide profile of individual organs and contributing to the functional diversity of ceramide species [1,6].
- Desaturation: In the final step, dihydroceramide desaturases (DES1 and DES2) introduce a trans double bond at the C4–C5 position of the sphingoid backbone, converting dihydroceramides (dhCer) into ceramides. This reaction determines the saturation state of the sphingoid base and regulates the intracellular balance between dhCer and ceramide [49]. DES1 is present broadly across tissues, whereas DES2 shows a more restricted distribution, particularly in the skin and intestine, where it also contributes to phytoceramides synthesis. This desaturation step is functionally critical, as ceramides—unlike their dhCer precursors—exert potent signaling and pro-apoptotic activities that influence multiple cellular processes [6,24].

Overall, the de novo pathway not only sustains basal sphingolipid synthesis but also links nutrient availability to lipid signaling and cellular homeostasis, with direct implications for physiology and disease [6]. After its synthesis in the ER, Cer must be transported to the Golgi apparatus for the generation of SM and glycosphingolipids [1,24,41].

Ceramide transport occurs through two main mechanisms. Non-vesicular transport, mediated by the ceramide transfer protein (CERT), is highly regulated and shows selectivity for long-chain ceramides. In parallel, Coat protein complexes I-dependent vesicular transport delivers ceramide to the *cis*-Golgi, where it serves as a substrate for glucosylceramide synthesis [1,3,24,41]. In addition, the hydrophobic nature of ceramides allows flip-flop movement across the lipid bilayer, ensuring luminal availability for downstream biosynthetic enzymes. Together, these mechanisms integrate ceramide trafficking with the maintenance and diversification of cellular sphingolipids pools [23].

#### 3.4.2. Hydrolysis of Sphingomyelin

SM hydrolysis is an essential catabolic pathway for the rapid generation of ceramide from the most abundant sphingolipid in the plasma membrane and endolysosomal system [50]. This process is catalyzed by the family of sphingomyelinases (SMases), which release ceramide and phosphocholine, and activated by stress stimuli—including inflammatory cytokines, radiation, reactive oxygen species (ROS), or infections—mobilizes the large SM reservoirs of the membrane, positioning this pathway as an immediate cellular defense system, in contrast to de novo synthesis, which is slower and more biosynthetic in nature [48,51]. SMases are classified according to their optimal pH as acidic, neutral, or alkaline [10,52].

Acid sphingomyelinase (aSMase, gen *SMPD1*) generates two isoforms: the lysosomal form (L-aSMase), which acts in the constitutive recycling of endocytosed SM, and the secretory form (S-aSMase), released into the extracellular space and zinc-dependent, both of which are involved in stress-induced signaling and various pathologies [51,53,54].

Neutral sphingomyelinase (nSMase) includes several isoforms, among which nSMase2 stands out. It is located in the plasma membrane and Golgi apparatus, regulated by phosphatidylserine, phosphorylation, and stress kinases, and activated by cytokines such as TNF-α and IL-1β, making it a key sensor of inflammation [50,55,56,57]. Finally, alkaline sphingomyelinase (alkSMase), which is confined to the intestine and bile, primarily participates in the digestion of dietary SM [34,52].

Overall, the sphingomyelinase pathway exemplifies the principle of lipid compartmentalization, generating specific ceramide pools in different organelles and contexts, which explains its functional versatility. Its dysregulation is associated with a wide spectrum of human diseases, including lysosomal disorders, neurodegeneration, cardiovascular pathologies, and cancer [51,58,59,60,61].

#### 3.4.3. Sphingosine Recycling

Ceramide derived from complex sphingolipid hydrolysis can undergo deacylation, in which the fatty acid bound by an amide linkage is removed, yielding Sph and a free fatty acid (FFA) [10]. This reaction is catalyzed by the family of ceramidases (CDases), of which five isoforms have been characterized, classified according to their optimal pH into acidic (aCDase), neutral (nCDase), and alkaline (alkCDases 1–3) [3,10].

The resulting sphingosine corresponds to a long-chain base (LCB) with amphiphilic properties that confer some solubility in the cytosol and the ability to transfer between membranes [1]. Once formed, it is transported to the ER, where it is reused in the synthesis of new ceramide through N-acylation catalyzed by Cer, an essential step that reincorporates the recycled Sph into the complex sphingolipid biosynthesis cycle [10]. Alternatively, Sph can be phosphorylated by sphingosine kinases (SphK1 and SphK2) to generate sphingosine-1-phosphate (S1P) [10].

This metabolite can subsequently be dephosphorylated in the ER by the specific phosphatases SPP1 and SPP2, regenerating Sph [3] which becomes available again for the CerS-mediated Cer resynthesis, thus completing the salvage cycle. It is worth noting that the regulation of SPP1 modulates the metabolic fate of S1P, favoring its channeling towards Cer regeneration [30,34].

### 3.5. Ceramide Synthase Enzymes

A key step in ceramide synthesis is the N-acylation of the long-chain sphingosine base by sphingosine N-acyltransferase, a reaction catalyzed in mammals by a family of six enzymes called ceramide synthases, encoded by genes previously known as LASS (Longevity Assurance) [26]. Although all catalyze the same reaction, each CerS displays marked specificity for the acyl-CoA chain length, thereby determining the fatty acid composition of the synthesized ceramides (Table 1) [26].

The degree of unsaturation of the N-acyl chain in ceramides is determined by the composition of the cytosolic acyl-CoA pool, which in turn is shaped by the coordinated actions of desaturases and elongases [65,66]. Among these enzymes, stearoyl-CoA desaturase-1 (SCD1) plays a central role by introducing a cis double bond at the Δ9 position of saturated fatty acids such as palmitoyl-CoA (C16:0) and stearoyl-CoA (C18:0), generating palmitoleoyl-CoA (C16:1) and oleoyl-CoA (C18:1), respectively [67]. These monounsaturated acyl-CoAs then serve as substrates for elongases, including ELOVL1 and ELOVL3, which extend the fatty-acyl chain in two-carbon increments to produce very-long-chain monounsaturated species such as C24:1 (nervonic acid) [66].

CerS2 Utilizes both lignoceroyl-CoA (C24:0) and nervonoyl-CoA (C24:1) to acylate the sphingoid base and generate very-long-chain ceramides. Consequently, the intracellular C24:0:C24:1 ratio reflects the relative activities of SCD1 and the elongases, serving as a functional index of cellular desaturation [67]. Although CerS contain structural determinants that shape their substrate specificity, the availability of acyl-CoAs ultimately governs whether saturated or unsaturated chains are incorporated [68].

Molecular studies using chimeric proteins have shown that an 11-amino-acid loop located between the last two transmembrane domains of CerS2 and CerS5 acts as a size filter that determines the preference for long acyl chains. Insertion of the CerS2 loop into CerS5 confers the ability to generate C22:0–C24:0 ceramides, demonstrating that the active-site architecture is modular and that the acceptance of C24:0 versus C24:1 depends on steric accessibility regulated by this domain [68].

Evidence from animal models supports this mechanism. SCD1-deficient mice display a marked reduction in monounsaturated ceramide synthesis and exhibit suppression of SPT activity, suggesting a feedback loop in which the accumulation of unprocessed saturated fatty acids limits sphingolipid biosynthesis [65]. Deep lipidomic analyses in CerS2-null mice show that loss of SCD1 results in complex remodeling of the ceramide profile, with reduced levels of VLCCs and increased LCCs, confirming that acyl-CoA availability and SCD1 activity are key determinants of the final sphingolipid composition [67].

The presence or absence of a double bond in the N-acyl chain of ceramides leads to profound biophysical differences in the cell membrane environment. Saturated ceramides generally exhibit high phase transition temperatures (T_m_) and pack tightly with cholesterol and saturated phospholipids [31,69]. Their linear conformation enhances Van der Waals interactions and promotes the formation of highly ordered domains, which stabilize the bilayer and reduces its permeability. In the case of VLCC, this extended geometry can even enable interdigitation between hemilayers, mechanically coupling both leaflets of the membrane and facilitating the emergence of gel phases ($L_{\beta }$) at physiological temperatures [31]. These properties are critical for barrier function and for maintaining the structural integrity of specialized membranes, such as those of the SC [37].

By contrast, unsaturated ceramides introduce a steric kink in the hydrocarbon chain that disrupts tight packing [31,69]. The *cis* double bond reduces chain alignment and lowers the melting temperature, thereby preventing the formation of rigid domains and favoring segregation into disordered liquid phases (L_d_). Moreover, unsaturation limits interdigitation between leaflets, resulting in membranes that are more fluid and permeable and less prone to stabilizing long-lasting lipid platforms [69]. This translates into several implications at the tissue level. The liver is the main producer of VLCCs due to the high expression of CerS2, maintaining a delicate balance between saturated and unsaturated species under physiological conditions. Models of acetaminophen-induced hepatotoxicity shift this balance toward saturated ceramides, which are associated with increased membrane rigidity and cellular dysfunction [70].

In contrast, during liver regeneration, unsaturated ceramides predominate, providing greater membrane fluidity and facilitating structural reorganization [67]. In the brain, the abundance of very long-chain sphingolipids is essential for myelin compaction. The rigidity conferred by saturated species ensures proper insulating function, whereas their loss as observed in CerS2 deletion models, leads to substitution by shorter or unsaturated chains. These species are unable to maintain structural integrity, ultimately resulting in severe neurodegeneration [67,71].

In the heart, the coexistence of saturated and unsaturated ceramides reflects a form of local adaptive regulation. Saturated ceramides contribute to membrane stability and energy metabolism, while in HF an increase in unsaturated ceramides is observed, likely as a response to hemodynamic stress, although with detrimental consequences for contractility [72].

### 3.6. Cellular Metabolism and Toxicity of Free Fatty Acid

FFAs are amphipathic molecules that can disrupt cell membranes when present at high concentrations, behaving similarly to detergents. This property makes excess FFAs potentially toxic to cells. To avoid lipotoxicity, cells tightly regulate intracellular FFA levels and maintain them at very low concentrations [73].

#### 3.6.1. Activation

After entering the cell, fatty acids are activated through their binding to coenzyme A (CoA), forming acyl-CoA molecules. This activation step is essential, as it prepares fatty acids for subsequent metabolism processes [4,74].

#### 3.6.2. Metabolic Destination

The metabolic fate of acyl-CoAs depends on the cell’s energy requirements.

- High energy demand:
  Acyl-CoAs are transferred to carnitine by carnitine palmitoyltransferase 1 CPT1 and transported into the mitochondria. There, they undergo β-oxidation to generate energy [4,75].
- Low energy demand:
  Acyl-CoAs are esterified with glycerol-3-phosphate to form triglycerides (TAG). These are stored in lipid droplets, which serve as the main cellular energy reserve [4,76]

#### 3.6.3. Excess Pathway

When cellular energy needs are satisfied and TAG storage capacity is exceeded, excess acyl-CoA is redirected toward Cer synthesis [4,74].

### 3.7. Physiological Functions of Ceramides

**Figure 3:**
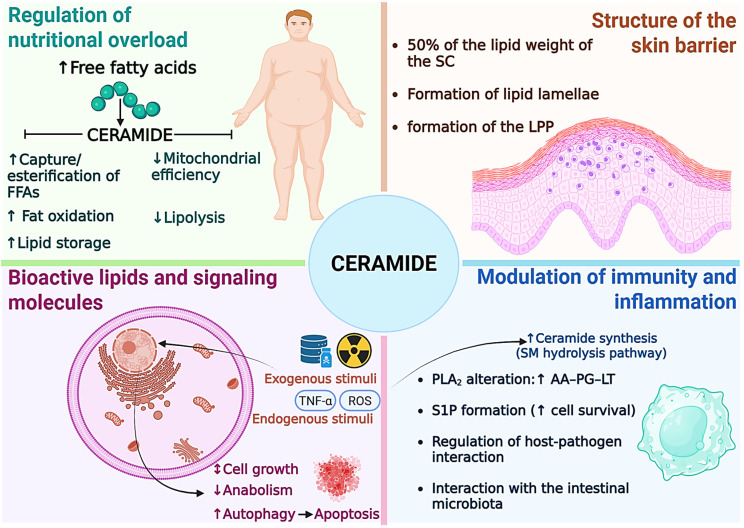
Physiological roles of ceramide. Ceramides integrate metabolic, structural, signaling, and immune functions. They promote adaptation to lipid overload, preserve skin barrier integrity, mediate stress-induced cellular responses, and regulate inflammatory processes. SC, stratum corneum; LPP, long periodicity phase; TNF-α, tumor necrosis factor alpha; ROS, reactive oxygen species; S1P, sphingosine-1-phosphate; PLA_2_, phospholipase A_2_; AA, arachidonic acid; PG, prostaglandins; LT, leukotrienes.

Each Cer species fulfills specific biological roles, which are summarized in Figure 3. Broadly, ceramides can be classified into four functional categories: (1) regulators of nutritional overload, (2) structural components of membranes, (3) bioactive signaling molecules, and (4) modulators of immunity and inflammation.

#### 3.7.1. Regulators of Nutritional Overload

Ceramides are evolutionarily conserved signals of lipid excess [77]. During nutritional overload, their accumulation initiates coordinated responses that aim to reduce FFA toxicity and preserve metabolic balance [4]. The main mechanisms include the following:

- Facilitation of safe uptake and esterification: Cer promote the translocation of the fatty acid transporter CD36 to the plasma membrane, thereby accelerating the uptake of extracellular FFAs. This process occurs in a controlled manner, allowing their immediate conversion into acyl-CoA by acyl-CoA synthetase, thus preventing the cytotoxic accumulation of FFAs [4,6,77].
- Promotion of lipid storage: The accumulation of Cer activates transcription factors such as Sterol Response Element Binding Proteins (SREBP), which increase the expression of genes involved in the synthesis of TAG and phospholipids [4,6,77]. As a result, cells channel excess fat into lipid droplets, promoting metabolically inert storage and reducing lipotoxicity.
- Induction of preferential fat utilization: Ceramides inhibit the uptake of glucose and amino acids, shifting energy metabolism toward fatty acid oxidation [4,6,77]. This change in fuel preference limits the availability of glycolytic substrates and imposes a lipid dependency that constitutes a molecular basis for the insulin resistance observed in obesity and T2DM [9,78].
- Reduction of mitochondrial efficiency: Cer interferes with the mitochondrial electron transport chain, reducing oxidative phosphorylation efficiency of and ATP production [4,6,77,79]. This phenomenon compels the cell to oxidize more fatty acids to meet its energy demands, generating an effect of dissipating excess energy as heat. Structurally, they promote DRP1-dependent mitochondrial fission, which reduces metabolic efficiency but helps alleviate lipid overload.
- Inhibition of the release of stored fats: Cer block lipolysis by inhibiting hormone-sensitive lipase (HSL) [4,6,77]. This mechanism prevents the further release of FFA into the cytoplasm, thereby avoiding a worsening of intracellular lipid overload.

Together, these mechanisms form an adaptative response that initially protects cells from lipotoxicity. However, when sustained over time, this program contributes to metabolic dysfunction and insulin resistance.

#### 3.7.2. Structural Components of Membranes and the Skin Barrier

The structural role of ceramides is most evident in the skin, where they account for approximately 50% of the lipid content of the SC [80]. Along with cholesterol (about 25%) and FFAs (10–15%), ceramides form an intercellular lipid matrix that serves as the body’s primary permeability barrier [80,81]. 

According to the classic “brick and mortar” model, corneocytes function as the bricks while the extracellular lipid matrix acts as the mortar that holds them together [82]. Ceramides present in this matrix are not synthesized directly in the SC. Instead, they originate from lipid precursors, such as glucosylceramides, produced by keratinocytes in deeper epidermal layers. These precursors are packaged into lamellar bodies and secreted into the extracellular space [80,82]. Once released, lipid precursors are hydrolyzed by specific enzymes that convert them into Cer, cholesterol, and functional FFAs. These components self-assemble into ordered sheets called lipid lamellae [80,81]. 

Barrier function depends strongly on ceramides containing very-long-chain fatty acids (C24:0–C26:0) and ultra-long-chain fatty acids (>C26:0–36:0) [81,83]. These long-chain species are essential for the formation of the long periodicity phase (LPP), a dense and stable lamellar arrangement that provides water impermeability and protection against external insults [29,81,84]. The length of the ceramide acyl chain strongly influences membrane properties. For example, C24:0 Cer form rigid and ordered phases that support barrier stability, whereas C16:0 Cer generate more fluid and disorganized structures, compromising barrier function [45,85,86].

#### 3.7.3. Bioactive Lipids and Signaling Molecules

In addition to their structural roles, ceramides act as bioactive signaling molecules involved in cellular stress responses. Their accumulation is commonly observed after exposure to external stressors, such as ultraviolet radiation and chemotherapy, as well as internal stress signals, including cytokines, oxidative stress, and DNA damage. Leading to their characterization as tumor-suppressor lipids [87,88].

Ceramides promote apoptosis, senescence, and cell-cycle arrest by inhibiting protein kinase B (Akt/PKB), a central kinase that supports cell survival and anabolic metabolism [89]. This inhibition occurs through the activation of protein phosphatase 2A (PP2A) and protein kinase Cζ, which deactivate Akt signaling and shift the cell toward a catabolic, energy-conserving state [89]. 

Cer also regulates autophagy, a cellular degradation process with dual outcomes. Depending on the Cer species involved and the cellular context, autophagy may promote survival during transient stress or lead to programmed cell death when damage is irreversible [87,88].

#### 3.7.4. Modulation of Immunity and Inflammation

Ceramides play a key role in regulating immune and inflammatory responses [4]. Pro-inflammatory cytokines, such as TNF-α, stimulate their production through the activation of SMases [4,89].

This process creates a positive feedback loop in which inflammation increases Cer levels, and ceramides further amplify cellular dysfunction and death, thereby sustaining tissue damage [6]. Ceramides also influence the production of inflammatory lipid mediators by organizing membrane microdomains that modulate phospholipase A_2_ (PLA_2_) activity. This enzyme releases arachidonic acid, the precursor of prostaglandins and leukotrienes [90].

As intracellular messengers, ceramides affect major signaling pathways, including NF-κB and mitogen-activated protein kinases (MAPKs) [90,91]. However, immune regulation cannot be understood by evaluating ceramides in isolation. Instead, it reflects the dynamic balance of the entire sphingolipid metabolic network [91].

While ceramides are generally associated with pro-inflammatory and -apoptotic effects, their metabolite S1P exerts opposing actions, promoting cell survival and lymphocyte trafficking [90,91]. The balance between ceramide and S1P, known as the ceramide/S1P rheostat, is a critical determinant of cell fate [88,91].

Elevated Cer synthesis may promote inflammation if ceramides accumulate, but not if they are rapidly converted to S1P [89]. This metabolic balance is also relevant in host–pathogen interactions, as some microorganisms manipulate ceramide metabolism to enhance colonization, while sphingolipids derived from the gut microbiota contribute to immune homeostasis [91].

### 3.8. Detection and Quantification of Ceramides

Liquid chromatography coupled to mass spectrometry (LC-MS) is widely recognized as the gold standard for lipid analysis [92]. Unlike immunochemical assays, which suffer from cross-reactivity and lack molecular specificity, LC-MS can resolve individual ceramide species that differ by acyl-chain length or degree of unsaturation, an essential requirement for precision medicine applications, including cardiovascular risk prediction based on ceramide profiles [93,94,95].

Among LC platforms, reversed-phase liquid chromatography (RPLC) predominates in clinical workflows, employing hydrophobic C8/C18 stationary phases and organic mobile-phase gradients to separate species largely according to hydrophobicity. As a result, LCCs (e.g., C16:0 Cer) elute before VLCCs (e.g., C24:0 Cer), and unsaturated species elute before their saturated counterparts [92,93,94,96]. Driven by clinical demand, high-throughput RPLC methods using short C18 columns and rapid gradients now enable quantification of up to 26 ceramide and dihydroceramide species in <5 min [93].

Although less common in clinical settings, normal-phase LC (NPLC) remains valuable for profiling entire lipid subclasses and poorly soluble, highly hydrophobic species. For example, NPLC using a polyvinyl-alcohol column and a heptane/isopropanol/ethanol gradient enabled separation of SC ceramides with up to 76 total carbon atoms is useful for characterizing the lamellar structures underpinning skin barrier integrity [97].

While LC-MS offers quantitative accuracy, it lacks spatial resolution. High-resolution MALDI imaging mass spectrometry (MALDI-IMS FTICR) has emerged as a complementary approach to visualize sphingolipid distribution directly in tissues. In situ enzymatic digestion with recombinant CDases or SMases enhances detection of low-abundance species and allows structural confirmation within defined microenvironments, such as tumor regions in lung carcinoma or pathological lipid deposits in Farber disease models [98].

In contrast, antibody-based assays face substantial specificity limitations. Early IgM monoclonal anti-ceramide antibodies showed marked cross-reactivity with SM and other phospholipids, precluding reliable analytical use [99]. Later, rabbit polyclonal IgG antibodies with higher affinity for the ceramide backbone offered improved specificity and enabled visualization of subcellular pools, including Golgi-associated ceramide, in cell biology applications [100]. Nonetheless, these antibodies cannot distinguish individual molecular species and therefore cannot substitute for LC-MS.

Enzyme-Linked Immunosorbent Assay (ELISA)-based detection of anti-ceramide antibodies has shown clinical relevance in selected contexts. In multibacillary leprosy, serum anti-ceramide IgM levels were significantly higher than in paucibacillary cases or controls (0.559 ± 0.396 vs. 0.302 ± 0.172 and 0.148 ± 0.084 OD units), with an AUC of 0.984 and near-perfect sensitivity/specificity [101]. Similarly, obstructive sleep apnea has been associated with elevated anti-ceramide antibodies (median 318 vs. 247.7 ng/mL, *p* < 0.0001) and decreased S1P concentrations (573.9 vs. 1006 ng/mL, *p* < 0.0001), reflecting perturbations in the ceramide/S1P axis and an independent association with endothelial dysfunction [102]. These findings highlight the utility of antibody-based assays as indirect biomarkers, albeit not as substitutes for molecular quantification.

Finally, to resolve subcellular ceramide pools, innovative workflows apply bacterial CDases to fixed cells, selectively hydrolyzing plasma-membrane ceramide while preserving intracellular stores [103]. This approach allows for the selective hydrolysis of ceramide located in the plasma membrane, without affecting the abundant intracellular reservoir. Hydrolysis converts membrane ceramide to sphingosine, which is subsequently quantified by mass spectrometry, providing a direct and specific measure of this pool.

This allows for the differentiation of the plasma compartment from the rest of the cell and demonstrates that plasma membrane ceramide constitutes a stable reservoir for at least one hour before being metabolized to sphingomyelin, glycosphingolipids, or S1P [103]

## 4. Evidence of the Role of Ceramides in Metabolic and Cardiovascular Disease

The accumulation of bioactive lipids in non-adipose tissues, including the heart, liver, and skeletal muscle, promotes cellular dysfunction known as lipotoxicity. This process contributes to the development of major cardiometabolic disorders such as MASLD, T2DM, CKD, CAD, MI and HF [6,77,104]. Among these lipids, ceramides play a central role by modulating intracellular signaling pathways that are critical for metabolic and cardiovascular homeostasis.

At the cellular level, Cer interfere with insulin signaling by reducing Akt/PKB phosphorylation, a key step required for GLUT4 translocation and glucose uptake [105]. In cardiac tissue, Cer contribute to myocardial fibrosis through regulation of collagen gene expression via regulated intramembrane proteolysis [77], and promote cardiomyocyte apoptosis by increasing mitochondrial outer membrane permeabilization (MOMP), leading to cytochrome *c* release and caspase activation [4,106]. These mechanisms are directly relevant to adverse cardiac remodeling and heart failure progression.

**Figure 4:**
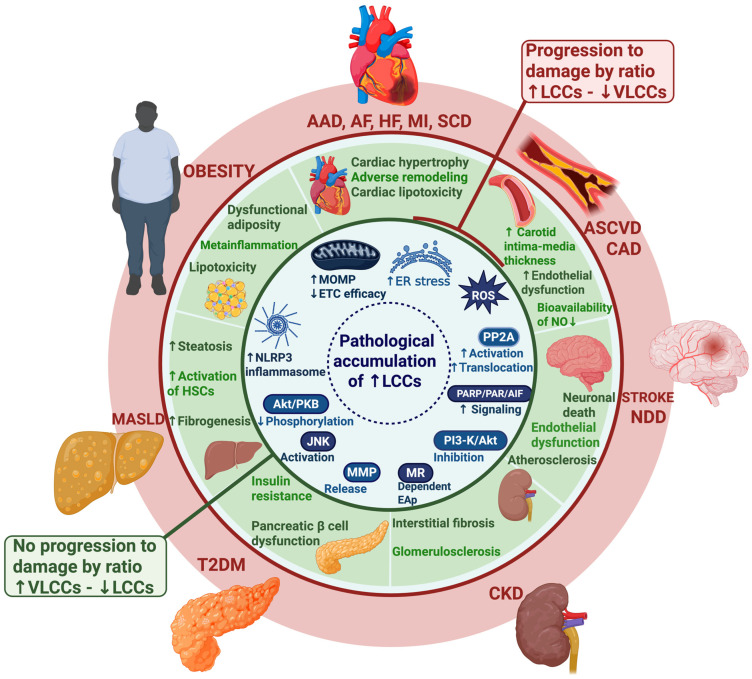
Ceramide (Cer) as a central axis in the progression of metabolic diseases, organized into three levels. Molecular level (blue circle): Pathological accumulation of long-chain ceramides (LCCs) triggers mitochondrial dysfunction (↑ MOMP, ↓ ETC), ER stress, ROS generation, and altered signaling (↓ AKT/PKB, ↑ JNK, ↑ MMP, ↑ PP2A, ↑ PARP/PARP-1, ↓ PI3K/AKT), leading to inflammation, apoptosis, and matrix degradation. Pathophysiological level (green circle): When LCCs are not counterbalanced by very-long-chain ceramides (VLCCs), cellular stress manifests as insulin resistance, endothelial dysfunction, lipotoxicity, oxidative stress, and tissue remodeling. Clinical level (red circle): These cascades contribute to obesity, MASLD, T2DM, CKD, NDDs, stroke, ASCVD/CAD, AAD, AF, HF, MI, and SCD. Ceramide thus links molecular disruptions to systemic disease progression.

In the vascular system, specific Cer species promote endothelial dysfunction, vascular cell apoptosis, and subendothelial retention of atherogenic lipoproteins, processes that are central to atherosclerosis development and progression [107,108,109]. Importantly, accumulating evidence indicates a structure–function relationship, in which LCCs such as C16:0 Cer are consistently associated with lipotoxicity and adverse cardiovascular effects, whereas VLCCs such as C24:0 Cer display neutral or potentially protective associations [30,48,105]. These findings provide a mechanistic basis for the use of circulating ceramides as clinically relevant biomarkers. Figure 4 shows the Cer as a central axis in the progression of metabolic diseases.

Based on these mechanistic and observational findings, several groups have developed ceramide-based lipid scores aimed at improving cardiovascular risk stratification in both secondary and primary prevention settings. The first model, CERT1, was constructed using three ceramides —Cer(d18:1/16:0), Cer(d18:1/18:0), and Cer(d18:1/24:1)— along with their ratios, and demonstrated a robust association with cardiovascular mortality in patients with established CAD [95].

Subsequently, the CERT2 score expanded this approach by incorporating phosphatidylcholines—PC(14:0/22:6), PC(16:0/22:5), and PC(16:0/16:0)—in addition to ceramides, generating a composite score ranging from 0 to 12 based on population quartiles [110]. This score was validated across multiple cohorts, including WECAC, LIPID, KAROLA, and FINRISK, and showed a progressive increase in cardiovascular mortality risk with higher scores. Patients in the highest category (≥9 points) exhibited a 3.5 to 5.4-fold increased risk in secondary prevention and an even greater risk (>10-fold) when combined with high-sensitivity troponin [110]. In primary prevention cohorts such as FINRISK 2002, CERT2 retained predictive value independent of conventional lipids parameters, supporting its potential clinical utility for early risk identification and prevention strategies. CERT 2 score defined risk categories (0–3 low, 4–6 intermediate, 7–8 increased, 9–12 high) [17,110,111,112].

The degree of unsaturation and the structural diversity of ceramides are key determinants of their biological behavior and their association with CVD [30]. The introduction of double bonds alters fundamental biophysical properties, including fluidity, lipid packing and membrane domain organization resulting in species, chain length and context-dependent effects. In endothelial cells, these properties are essential for mechano-transduction, NO production, and blood pressure regulation [113].

Recent studies indicate that unsaturation can shape both protective and adverse cardiometabolic profiles [95], in part by modifying the interaction of ceramides with signaling proteins and membrane microdomains and thereby influencing pathways such as Akt/eNOS [114]. Beyond canonical species, less abundant variants, such as ceramides with irregular chain lengths, sphingoid bases containing multiple double bonds (e.g., d18:2), and polyunsaturated acyl chains, add further structural complexity [115]. Although typically present at low physiological levels, these species may gain relevance in pathological settings by finely modulating membrane architecture or serving as sensitive biomarkers of metabolic dysfunction [115].

Experimental evidence shows that d18:2-based lipids accumulate in Sphingosine Kinase 2 (SphK2)-deficient models, suggesting slower metabolism and potential disruption of lipid homeostasis, with downstream pro-apoptotic and pro-inflammatory consequences in vascular and cardiac cells [114,115]. Finally, the biosynthetic and catabolic pathways that include desaturases, elongases, CerS enzymes, and lysosomal hydrolases determine the abundance and fate of these diverse species [116]. Disruption of these pathways not only alters lipid composition but also affects cell signaling, modulating Akt/eNOS activity, ROS production, and pro-inflammatory cascades and interferes with the formation of functional membrane domains, thereby linking sphingolipid biochemistry to cardiovascular pathophysiology [30,47,113].

The current evidence on the most extensively studied ceramides in cardiovascular disease is summarized in the following sections. For clarity, the pioneering studies that supported the development of ceramide-based risk scores are presented in Table 2.

Throughout this manuscript, ceramides are referred to by the number of carbons and double bonds in the acyl chain, assuming the standard sphingoid base d18:1, in order to simplify nomenclature and enhance clarity of presentation.

### 4.1. Ceramide 14

Ceramide C14:0 is derived from myristic acid, a 14-carbon saturated fatty acid, and is primarily synthesized by CerS6 [119]. CerS6 expression is increased in obesity and has been implicated in metabolic disturbances affecting adipose and hepatic tissue [26,47]. Evidence summarized in Table 3 suggests that C14:0 Cer does not function as a primary initiator of lipotoxicity but rather acts as an amplifier of preexisting lipotoxic signals [119,120].

In an in vitro study, Martínez et al. [120] evaluated the effects of palmitic acid (PA), myristic acid (MA), and their combination in primary mouse hepatocytes. While MA alone was not lipotoxic, co-exposure with PA significantly increased lipoapoptosis compared to PA alone (*p* < 0.05). Notably, the combination of 0.5 mM PA and 0.5 mM MA induced a degree of cell death comparable to that observed with 1.0 mM PA. This synergistic lipotoxic was associated with increased caspase-3 activation and cytochrome c release, supporting a pro-apoptotic mechanism. These findings suggest that the toxicity effects of C14:0 Cer are conditional and require concurrent lipotoxic stress, such as palmitate exposure.

Within the context of metabolic syndrome (MS), where multiple fatty acids coexist, C14:0 Cer may function as a signaling molecule that amplifies cellular stress and promotes progression toward apoptosis. Although these findings were initially described in hepatocytes, a similar mechanism is likely relevant to the cardiovascular system. Excess fatty acid availability may induce C14:0 Cer accumulation in cardiomyocytes, contributing to the lipotoxic cardiomyopathy [120].

Supporting this concept, Russo et al. [121] demonstrated in murine and in vitro models of diabetic cardiomyopathy that upregulation of CerS5 was associated with increased C14:0 Cer levels and cardiomyocyte hypertrophy mediated by autophagy activation. In isolated cardiomyocytes, myristate treatment increased C14:0 Cer concentrations approximately 10-fold and was accompanied by a significant increase in cell size, whereas palmitate had no effect. Mechanistically, silencing of LC3B prevented myristate-induced hypertrophy, and myristate exposure significantly increased the expression of autophagy-related genes (Becn1 and Atg7) as well as GFP-LC3B-II and free GFP signals, indicating enhanced autophagic flux (*p* < 0.05). Importantly, silencing of CerS5 via siRNA (small interfering RNA) completely prevented MA-induced hypertrophy. Although CerS5 is classically linked to C16:0 Cer synthesis, these findings suggest that, in the cardiac context, CerS5 may also contribute to C14:0 Cer production, a hypothesis that warrants further investigation [121].

At the clinical level, the role of C14:0 Cer is less well established than that of other ceramide species. Although it has been evaluated in cardiometabolic studies [47], C14:0 Cer is not commonly included as a primary marker in cardiovascular risk panels. Population studies have reported higher baseline levels of C14:0 Cer in healthy older adults compared with younger individuals [18], suggesting that its presence may not directly reflect pathological risk.

Consistent with this observation, prognostic studies have not demonstrated clear predictive value for C14:0 ceramide when measured in isolation. In patients with MI, de Carvalho et al. [122] reported no statistically significant association between C14:0 Cer levels and MACE. Accordingly, C14:0 Cer is not included in validated prognostic scores such as CERT1 or CERT2, which rely on other Cer species to predict adverse cardiovascular outcomes [122]. This omission highlights an important knowledge gap between mechanistic evidence and clinical utility.

Additional insights come from studies in advanced heart failure and metabolic intervention. In patients with end-stage HF, Ruiping Ji et al. [13] reported a 1.6-fold increase in LCCs and VLCCs in the myocardial tissue, which was partially reversible following after mechanical unloading with a left ventricular assist device (VAD). Interestingly, serum ceramide profiles showed an opposite pattern: while damage-associated ceramides decreased after recovery, C14:0 and C24:0 Cer species increased by 33% and 44%, respectively. This divergence between tissue and circulating levels suggests that circulating C14:0 Cer may reflect adaptive or reparative processes rather than a direct myocardial injury in this context.

In a metabolic setting, Kasumov et al. [123] demonstrated that 12 weeks of high-intensity aerobic training significantly reduced total plasma ceramides and specifically decreased C14:0 ceramide levels (*p* < 0.007) in adults with obesity, with or without T2DM. The reduction in C14:0 ceramide was strongly correlated with improvements in peripheral insulin sensitivity, assessed by glucose infusion rate, and C14:0 ceramide emerged as the strongest predictor of this metabolic improvement (r = −0.56, *p* = 0.009). [123]. Collectively, current evidence suggests that C14:0 ceramide functions as a context-dependent amplifier of lipotoxicity rather than an independent driver of cardiometabolic disease.

### 4.2. Ceramide 16

C16:0 ceramide, derived from palmitic acid, is primarily synthesized by CerS5 and CerS6 [26,124]. Nutritional overload increases palmitate availability and induces the overexpression of CerS5/6, resulting in the pathological accumulation of C16:0 Cer [78,125]. This lipid species acts as an intracellular signal of energy excess and cellular stress [126]. Multiple studies summarized in Table 4 identify C16:0 Cer as a potent pro-apoptotic mediator [6,78,88].

At the cellular level, C16:0 Cer alters membrane biophysical properties and promotes the formation of ceramide-rich platforms [88]. It interacts with pro-apoptotic BCL-2 family proteins, including BAX, facilitating their oligomerization and outer mitochondrial membrane permeabilization [88,127]. In addition, C16:0 Cer binds directly to VDAC2, enhancing pore formation and cytochrome *c* release, thereby activating the intrinsic caspase-dependent apoptosis pathway [6,88]. These mechanisms contribute to the loss of cardiomyocytes, hepatocytes, and endothelial cells, representing a key link between lipid overload and organ dysfunction [6,126,128].

**Table 4:** Summary of C16:0 Ceramide Mechanisms Across Experimental and Clinical Context.

Study Type (Reference)	Biological Matrix	Organelle Involved	Observed Pathophysiological Mechanism
In vitro [126]	Hepatocytes (primary)	ER + Mitoc + PM	PA → Palmitoyl-CoA → de novo synthesis → ↑ C16:0 Cer → accumulates in lipid rafts → clustering of TRAIL-R2 → DISC → caspase-8 ↑ → tBid → mitoc → formation of Cer channels that cooperate with Bax/Bak → MOMP → cytochrome c ↑ → caspases 3/6/7 ↑ → apoptosis
			ER stress (↑ CHOP) → ↑ PUMA/Bim → activates Bax/Bak → hepatocyte lipoapoptosis + release of pro-inflammatory EVs
In vitro [129]	Macrophages (primary/peritoneal, murine lines)	Lysosome + Mitoc+ ER	Signal 1: PA → ↑ C16:0 Cer → acts as DAMP → TLR4/CD36 ↑ → ↑ transcription Nlrp3, pro-IL1β, pro-IL18.
			Signal 2: C16:0 Cer → lysosomal destabilization (cathepsin B ↑) + mitochondrial ROS ↑ + K^+^ efflux → NLRP3-ASC-procaspase-1 assembly → active caspase-1 ↑ → pro-IL1β/IL18 cleavage → IL1β/IL18 secretion → chronic sterile inflammation → M1 polarization, metabolic inflammation, insulin resistance
In vitro [125]	Mice hepatocytes (C57BL/6J)	RE + Mitoc + PM	Palmitate (HFD) → ↑ CerS6 → ↑ C16:0 Cer → insertion into plasma membrane and mitochondria
			PM: C16:0 Cer forms lipid platforms → sequestration of IR/PI3K → ↓ phosphorylation of Akt/PKB → ↓ glucose uptake → Insulin resistance
	adipocytes		
			ER: lipid stress → ↑ TAG ↑ C16:0 Cer → inhibitory signal towards Akt and ↑ WAT inflammation
	BAT		
			Mitochondria: C16-Cer → ↓ Complexes II and IV of the ETC → ↓ β-oxidation → ↓ ATP synthesis + ↑ ROS → Mitoc dysfunction
In vivo	HFD in wild-type vs. CerS6 KO mice	RE + Mitoc	CerS6 KO → ↓ C16:0 Cer → ↑ β-oxidation (Mitoc) → ↑ energy expenditure → protection against DIO and insulin resistance
In vitro	Hepatocytes + BAT cells		Liver: ↑ Cd36, Fabp4, SCD1 → ↑ lipid uptake and storage → ↓ palmitate oxidation → hepatic insulin resistance
Ex vivo [130]	WAT, BAT, liver explants + human adipose biopsies		WAT: ↑ body weight ↑ inflammation ↑ serum leptin ↓ glucose tolerance ↓ Akt phosphorylation (↓ insulin signaling) BAT: ↓ lipid oxidation, ↑ accumulation of lipid droplets → ↓ energy expenditure → obesity
In vitro	Hepatocyte (mouse liver)	RE + Mitoc	↑ CerS6 → ↑ C16:0 Cer ↑ → ↓ β-oxidation → ↓ ATP → ↑ TAG → mitochondrial dysfunction → impaired FA oxidation → insulin resistance (↓ Akt/PKB-P)
In vivo	CerS2^+^/^−^ mice		CerS2^+^/^−^ → compensatory ↑ C16:0 Cer (via CerS6) → ↓ FA oxidation → acylcarnitine ↑ → steatosis → hepatocellular apoptosis (↑ cleaved PARP) → insulin resistance (↓ glucose tolerance)
In vitro [131]	Adenoviral CerS6 upregulation		CerS6↑ → ↑ C16:0 Cer → ↓ ETC complex II & IV→ ↓ O_2_ consumption → lipid droplet ↑ → insulin signaling inhibition (↓ Akt/PKB)
In vitro/ex vivo [132]	Human skeletal muscle (myocytes: vastus lateralis fibers)	RE *SS* (PM) *IMF* (Mitoc)	PA uptake → de novo synthesis of C16-Cer (ER) → preferential accumulation in the *SS* fraction → ↑ C16:0 Cer SS ↔ insulin ↑ ↔ HOMA-IR ↑
			*SS* C16:0 Cer → lipid platforms in PM → inhibition of IR/PI3K signaling → ↓ Akt/PKB phosphorylation, ↑ Muscle insulin resistance (↓ GLUT4 translocation, ↓ glucose uptake)
			*IMF* C16:0 Cer does not correlate with insulin resistance → localized effect in *SS*
In vitro/ex vivo [133]	C2C12 myotubes (murine) skeletal muscle fibers (red gastrocnemius)	Mitoc (fusion/fission dynamics)	PA → ↑ de novo Cer synthesis (SPT2) → ↑ C16:0 Cer → activates Drp1 ↑ → excessive mitochondrial fission → ↓ mitochondrial respiration (↓ O_2_ consumption, complex II) → ↑ ROS (H_2_O_2_) → ↓ phospho-Akt/PKB → muscle insulin resistance
			Inhibition of Drp1 (Mdivi-1) → blocks pathological fusion → protects maintained mitochondrial respiration, ↓ ROS, preserved insulin signal
In vitro ex vivo	HUVEC Human thoracic (ThAT) vs. subcutaneous (ScAT) adipose tissue	ER + Mitoc + PM	↑ CerS6 → ↑ C16:0 Cer secretion (via EVs) → uptake by endothelium ⟶ PERK→p-eIF2α→CHOP↑ ⟶ ER stress ⟶ ROS↑ ⟶ ↓ NO ⟶ endothelial dysfunction ⟶ insulin resistance.
			ThAT↑ C16:0 Cer (vs. ScAT) → ↑ Cer -enriched EVs ⟶ correlated with ↑ vascular O_2_ and HOMA-IR → vascular oxidative stress ⟶ metabolic impairment
In vivo (mouse) [134]	Plasma and vascular tissue		
			Adipose-derived EVs (C16:0 Cer+) ⟶ ↑ vascular ROS ⟶ ↓ insulin-mediated vasodilation ⟶ endothelial insulin resistance and redox imbalance
Clinical [20,44,110,135,136]	Serum/plasma	Systemic	↑ C16:0 Cer or ratios ↑ C16:0/↓ C24:0 Cer⟶ adverse ceramide remodeling (CerS imbalance) ⟶ strong independent predictor of MACE, CV mortality and HF; reflects cardiometabolic/multiorgan dysfunction (heart, liver, kidney) ⟶ correlates with ASCVD; prognostic risk ↑ with T2DM, mitigable by Mediterranean diet
			Inferred mechanism: C16:0 Cer↑ (plasma) ⟶ vascular/myocardial uptake ⟶ ER stress + mitochondrial dysfunction (ROS↑, ATP↓) ⟶ endothelial dysfunction (NO↓) + inflammation + apoptosis ⟶ atherosclerosis progression/MI/ventricular remodeling/arrhythmia ⟶ clinical events (MACE, MI, HF, SCD)

Cer, ceramide; PA, palmitic acid; ER, endoplasmic reticulum; PM, plasma membrane; Mitoc, mitochondria; SS, subsarcolemmal; IMF, intramyofibrillar; ETC, electron transport chain; EVs, extracellular vesicles; HF, heart failure; MI, myocardial infarction; MACE, major adverse cardiovascular events; T2DM, type 2 diabetes mellitus; CerS, ceramide synthase; SPT, serine palmitoyltransferase; SPTLC2, serine palmitoyltransferase long chain base subunit 2; IR, insulin resistance; TAG, triacylglycerol; ROS, reactive oxygen species; NO, nitric oxide; ↑: increase; ↓: decrease.

Beyond apoptosis, C16:0 Cer functions as a damage-associated molecular pattern (DAMP), activating innate immune receptors such as TLR4 and the NLRP3 inflammasome, particularly in macrophages [6,129,137]. Activation of NLRP3 promotes to the caspase-1 cleavage and the maturation of IL-1β and IL-18, directly linking lipid excess to chronic low-grade inflammation characteristic of atherosclerosis and insulin resistance [125,127,129]. 

C16:0 Cer also plays a central role in the development of insulin resistance. Its accumulation interferes with insulin receptor signaling through activation of PP2A, which dephosphorylates and inactivates Akt [6,9,78,125]. As a result, GLUT4 translocation in muscle and adipose tissue is impaired and hepatic gluconeogenesis is inadequately suppressed, leading to systemic insulin resistance.

Human studies provide strong translational support for these mechanisms. Turpin et al. [130] analyzed visceral and subcutaneous white adipose tissue from 439 subjects, demonstrating that CerS6 expression was positively associated with body mass index (BMI), body fat content, and hyperglycemia, and inversely associated with insulin sensitivity measured by euglycemic-hyperinsulinemic clamps. In a subcohort of individuals with obesity, visceral C16:0 Cer levels were markedly elevated compared with lean controls (*p* < 0.05) [130]. Causal evidence derives from murine genetic models. Hepatic deletion of CerS6 selectively reduced C16:0 Cer levels, preserved Akt phosphorylation in hepatocytes exposed to PA, and improved glucose tolerance (*p* < 0.05) [130].

Conversely, Raichur et al. [131] demonstrated that adenoviral overexpression of CerS6 increased C16:0 Cer accumulation (*p* < 0.05), inhibited Akt/PKB phosphorylation (*p* < 0.001), promoted TAG accumulation (*p* < 0.0001), and impaired mitochondrial function via inhibition of electron transport chain (ETC) complex II. Together, these findings establish CerS6-driven C16:0 Cer synthesis as a causal mediator of metabolic dysfunction.

The pathogenic effects of C16:0 Cer depend not only on its total concentration but also on its subcellular localization [88]. In skeletal muscle, subsarcolemmal (SS) ceramides, located near the plasma membrane, show a stronger association with insulin resistance. Chung et al. [132] demonstrated that the association between intramyocellular ceramides and insulin resistance is largely confined to the subsarcolemmal (SS) fraction, whereas intramyofibrillar (IMF) ceramides showed no correlation with metabolic parameters. This compartmental specificity proximity to insulin signaling complexes, enabling direct interference with receptor-mediated pathways. Long-chain SS ceramides (C16:0 and C18:0 Cer) were positively associated with markers of insulin resistance. The strongest correlation was observed for SS Cer C16:0 with HOMA-IR (r = 0.429, *p* = 0.0004) and plasma insulin (r = 0.413, *p* = 0.0006). Similarly, SS Cer C18:1 correlated with HOMA-IR (r = 0.359, *p* = 0.003) and plasma TAG (r = 0.389, *p* = 0.001), highlighting the importance of subcellular localization in the metabolic relevance of these species.

Accumulation of C16:0 Cer in the mitochondria alters cellular energy and promotes the production of ROS. Smith et al. [133] demonstrated that palmitate-induced C16:0 Cer accumulation promotes sustained mitochondrial fission through activation and upregulation of Drp1. In cultured myotubes and permeabilized muscle fibers, treatment with C16:0 Cer led to a significant reduction in oxygen consumption (*p* < 0.05), confirming its role in bioenergetic disruption. These findings suggest that therapeutic strategies targeting Cer accumulation in critical cellular compartments may be more effective than approaches aimed solely at reducing total ceramide levels.

Beyond metabolic dysregulation, C16:0 Cer is a central mediator in CVD [138]. It contributes to cardiomyocyte and endothelial dysfunction, atherosclerotic plaque formation, and clinical events such as MI and HF [108,114]. Atherosclerosis, the underlying process in most CVDs, is characterized by arterial narrowing due to plaque accumulation. C16:0 Cer contributes to its initiation and progression through mechanisms involving vascular endothelium and immune cells [108,138]. Under physiological conditions, the endothelium maintains vascular tone and prevents thrombosis through the production of nitric oxide (NO), synthesized by endothelial nitric oxide synthase (eNOS) [113]. Endothelial dysfunction induced by C16:0 Cer compromises this protective mechanism, promoting atherosclerotic progression.

Experimental studies demonstrate that exogenous C16:0 Cer directly induces vascular dysfunction [139]. In human aortic endothelial cells, Akawi et al. [134] demonstrated that C16:0 Cer increases superoxide production through the uncoupling of tetrahydrobiopterin-dependent eNOS, converting it from a NO-producing enzyme into a source of ROS, an early hallmark of endothelial dysfunction and atherosclerosis. In a clinical study, part of the same trial, each standard deviation increases in plasma C16:0 Cer was associated with a 1.4-fold higher risk of sudden cardiac death (SCD), and its glycated variant with a 1.6-fold higher risk, independent of other risk factors. Furthermore, treatment with liraglutide reduced these levels, suggesting that, although basal C16:0 may be involved in vasodilatory signaling, its chronic accumulation is pro-oxidative and harmful [134].

In the atherosclerotic context, C16:0 Cer acts as a DAMP, activating macrophages and inflammation via NLRP3 and IL-1β [129]. C16:0 Cer is directly toxic to cardiomyocytes [6]. As previously mentioned, its accumulation participates in the activation of intrinsic apoptosis through mitochondrial permeabilization [88,137]. This effect contributes to the loss of functional myocardium, adverse remodeling, and HF [6]. Elevated levels of C16:0 Cer have been found in patients with STEMI, correlated with plaque rupture and the severity of coronary stenosis [140]. In a community-based cohort followed for 13 years, individuals in the highest quartile of a ceramide score (including C16:0 Cer) had a 1.47-fold higher risk of MACE (MI, revascularization, stroke, or death; 95% CI: 1.12–1.92) [17].

In the TOPCAT trial, circulating C16:0 Cer was the species most strongly associated with death or hospitalization for HF (adjusted HR: 1.35; 95% CI: 1.13–1.63; *p* = 0.0011) [128]. In an analysis of the Framingham Offspring cohort, a higher proportion of C16:0 Cer was observed to be associated with adverse changes in cardiac structure and function, including lower left ventricular ejection fraction (LVEF), worse global circumferential strain (GCS) of the left ventricle, higher left atrial end-systolic volume (LAVes), and lower left atrial emptying fraction (LAEF) [136]. Additionally, increased plasma levels of C16:0 Cer have been demonstrated in patients who died from chronic HF [141]. In the same context, it has been associated with incident atrial fibrillation (AF) [95], coronary plaque vulnerability [142], endothelial dysfunction in DM [143] and increased carotid intima-media thickness [144].

Large cohort studies further support its prognostic relevance beyond conventional risk factors [95]. In a primary prevention context, in the PREDIMED trial, individuals in the highest quartile of baseline C16:0 Cer had a 2.39-fold higher risk of incident CVD (95% CI: 1.49–3.83; *p* < 0.001) [145].

The risk associated with C16:0 is better contextualized when compared with VLCCs. Meta-analyses from the Framingham Heart Study and SHIP cohorts demonstrated that ratios such as C24:0/C16:0 Cer outperform individual species in predicting cardiovascular risk [14,20]. Higher ratios were associated with a lower incidence of CAD (HR: 0.79; 95% CI: 0.71–0.89; *p* < 0.0001), HF hospitalization (HR: 0.78; 95% CI: 0.61–1.00; *p* = 0.046), and all-cause mortality (HR: 0.60; 95% CI: 0.56–0.65; *p* < 0.0001) [20]. The C24:0/C16:0 ratio serves as a robust biomarker because it captures the net balance between pro-pathogenic (C16:0 Cer) and homeostatic/protective (C24:0 Cer) effects of sphingolipid metabolism [20]. A low ratio indicates a system overwhelmed by lipotoxic stress signals (C16:0 Cer) relative to structural and potentially protective ceramides (C24:0 Cer) [146]. Therefore, the ratio not only reflects an excess of a harmful lipid but also a failure in compensatory mechanisms, providing a more comprehensive assessment of an individual’s metabolic health status [20,146].

These concepts are incorporated into clinical risk scores such as CERT1 and CERT2, which are already used to improve cardiovascular risk stratification beyond traditional metrics [95,112]. The CERT1, based on C16:0, C18:0, and C24:1 Cer, and their ratios with C24:0 Cer, has demonstrated prognostic utility in CAD [117]. The CERT2, which incorporates specific phosphatidylcholines, enhances stratification accuracy and broadens its applicability across different populations [110,112]. Overall, both scores represent emerging tools for prognostic assessment in CVD, with CERT2 demonstrating greater predictive performance than its predecessor [18,47,95,147].

Accumulated evidence from experimental models and large human cohorts establishes C16:0 Cer as a central mediator in the pathogenesis of metabolic and CVD. It is not merely a bystander or a correlative marker, but a causal actor that translates nutrient excess into cellular dysfunction and, ultimately, clinical disease [17].

### 4.3. Ceramide 18

C18:0 ceramide is primarily synthesized by CerS1 and CerS4, which incorporate stearic acid (C18:0) or, to a lesser extent, oleic acid (C18:1) as the acyl chain [21,26,124]. Accumulation of this Cer species has been associated with obesity and T2DM, sharing pathogenic mechanisms with C16:0 Cer, including inhibition of Akt/PKB signaling and mitochondrial dysfunction [18,30]. In addition to these shared pathways, the distinct biological and clinical roles of C18:0 Cer have been described; these are summarized in Table 5.

In the vascular context, C18:0 Cer has been implicated in endothelial injury mediated by mineralocorticoid signaling. Zhang et al. [148] demonstrated in human umbilical vein endothelial cells (HUVECs) that aldosterone exposure induces mineralocorticoid receptor (MR)-dependent apoptosis in a dose-dependent manner. These effects were accompanied by a 2–3-fold intracellular increase in C18:0 Cer, mediated by CerS1. Pharmacological blockade of the MR with eplerenone or inhibition of CerS1 abolished both ceramide accumulation and apoptosis (*p* < 0.05), establishing a direct aldosterone–MR–CerS1–C18:0 ceramide axis. These findings position C18:0 Cer as a mechanistic mediator of vascular damage relevant to hypertension and HF.

C18:0 Cer also plays a role in inflammatory vascular disorders. Yang et al. [149] reported significantly higher plasma levels of C18:0 Cer in patients with thoracic aortic dissection (TAD) compared to those with aneurysm (TAA) and controls (*p* < 0.001). In aortic tissue, upregulation of SPTLC2 and CerS6 in infiltrating macrophages (*p* < 0.01), indicated activation of de novo ceramide synthesis [149]. Experimental exposure of macrophages to C18:0 Cer increased the activation of the NLRP3 inflammasome, caspase-1, IL-1β, and MMP9 (*p* < 0.05–0.001), promoting extracellular matrix degradation and tissue rupture, mechanisms common to both atherosclerotic plaque vulnerability and TAD. In murine models of β-aminopropionitrile-induced TAD, inhibition of Cer synthesis with myriocin reduced inflammation and prevented dissection development (*p* < 0.05), supporting a causal role for C18:0 Cer in vascular wall destabilization.

At the metabolic level, elevated circulating C18:0 ceramide has been observed in individuals with metabolic syndrome. Lee et al. [150] reported significantly higher plasma concentrations of C18:0 Cer in affected subjects compared with healthy controls (65.2 ± 25.4 vs. 34.9 ± 12.9 ng/mL; *p* < 0.0001). Furthermore, genetic variants in *CerS3*, CerS6, *SPTLC2*, *SPTLC3*, *ACER1*, and *SGMS1* were significantly associated with increased levels of LCCs (*p* < 0.05). These findings suggest a genetic predisposition to C18:0 Cer accumulation and support its role in sustaining the lipotoxic and inflammatory milieu characteristic of MS.

Clinically, C18:0 ceramide has emerged as a robust prognostic biomarker. An increase Cer(C18:0)/Cer(C16:0) ratio predicts the incidence of T2DM [47], while elevated C18:0 Cer levels detectable up to nine years before diagnosis [142]. In patients with long-standing type 1 diabetes mellitus (T1DM), the (C18:0)/Cer(C24:0) ratio was associated with a higher risk of MACE and all-cause mortality, outperforming low-density lipoprotein cholesterol (LDL-C) in risk prediction [151].

Large cohort analyses further confirm these associations. Laaksonen et al. [44], in the combined analysis of three cohorts (Corogene, SPUM-ACS, and BECAC), confirmed that ceramide C18:0 Cer predicts cardiovascular mortality independently of LDL-C. In the SPUM-ACS cohort (*n* = 1637), each standard deviation increase in C18:0 ceramide was associated with a 66% higher risk of cardiovascular death after multivariable adjustment. Elevated C18:0 Cer has also been linked to adverse outcomes in ischemic stroke, underscoring its relevance across diverse vascular pathologies [152]

The prognostic relevance of C18:0 Cer extends beyond patients with established CAD, as associations with MACE have also been observed in primary prevention populations [145]. C18:0 Cer is a key component of the CERT1 and CERT2 risk scores, together with C16:0 and C24:1 Cer [95]. These indices improve cardiovascular risk stratification beyond traditional lipid measures and predict MI and cardiovascular death in both apparently healthy individuals and patients with CAD [95]. Collectively, current evidence establishes C18:0 ceramide as a clinically meaningful biomarker reflecting cellular stress, apoptosis, and vascular injury not captured by conventional lipid panels [152].

### 4.4. Ceramide 20

C20:0 Cer, which incorporates arachidic acid (C20:0) or gondoic acid (C20:1) asits acyl chain, is primarily synthesized by CerS2 and CerS4 [26,124]. Increasing evidence links this species to CVD and adverse metabolic outcomes [18]. Key studies supporting its biological and clinical relevance are summarized in Table 6.

Experimental data indicate that C20:0 Cer actively participates in pathological vascular remodeling. Zhang et al. [153] demonstrated that activation of the intestinal FXR–CerS2 axis promotes C20:0 Cer accumulation in a murine model of angiotensin II–induced aortic aneurysm and dissection. Plasma and tissue levels of C20:0 increased 2.5-fold higher than controls (*p* < 0.001), accompanied by an increase in the expression of CerS2 in intestinal epithelium and aortic macrophages, as well as exaggerated release of the metalloproteinases (MMP2 and MMP9) (*p* < 0.01). These enzymes degrade collagen and elastin, weakening the aortic wall and facilitating tissue rupture. Pharmacological activation of the intestinal Farnesoid X Receptor (FXR) with ursodeoxycholic acid significantly reduced the expression of CerS2 and C20:0 Cer levels (*p* < 0.001), resulting in a 58% decrease in aneurysm incidence and a 63% decrease in aortic dissection (*p* < 0.01). These findings establish a solid mechanistic link between C20:0 Cer accumulation and MMP-mediated extracellular matrix degradation and pointing to C20:0 Cer as a potential therapeutic target.

In the context of myocardial injury, Hadas et al. [154] reported a marked increase in in C20:0 Cer following experimental MI, with levels rising up to 16.6-fold in left ventricular tissue (*p* < 0.05). Modulation of ceramide metabolism through overexpression of acidic ceramidase reduced myocardial apoptosis, limited scar formation, and improved left ventricular ejection fraction at 28 days post-MI (*p* < 0.001). These findings suggest that C20:0 Cer contributes to early cardiomyocyte death and adverse remodeling, supporting its relevance as a mediator of post-infarction injury.

Clinical studies further support the prognostic value of C20:0 Cer. In patients with established CAD, Tarasov et al. [155] identified that the Cer(C20:0)/Cer(C24:0) ratio was significantly associated with cardiovascular mortality, showing that a 1 standard deviation increase corresponded to an adjusted OR of 1.50 (*p* = 0.0009), even after adjustment for LDL-C, BMI, and fasting glucose. While simvastatin reduced these Cer by approximately 25% and PCSK9 deficiency by 20%, ezetimibe showed no significant effect, suggesting that modulation of C20:0 Cer depends on the pharmacological pathway. These findings suggest that Cer are superior indicators compared to LDL-C for risk stratification of mortality in patients with CAD.

In myocardial tissue, ceramides C20:0 and C20:1 Cer are elevated in chronic ischemia and during MI, correlating with greater damage and ventricular dysfunction [107]. Similar increases have been reported in patients with HF [156].

The prognostic Cer signature derived from the study by Carvalho et al. [122] showed that C20:0 Cer was significantly associated with a higher risk of MACE in MI-patients, with the highest predictive capacity observed in measurements taken within 24 h following angiography. Plasma levels of C20:0 Cer decreased significantly from day 1 to day 2 (*p* < 0.05), possibly influenced by heparin administration during the invasive management of the MI. These findings support C20:0 Cer as a critical molecular mediator in pathological vascular remodeling and myocardial damage, as well as a prognostic biomarker potentially superior to traditional cardiovascular risk scores [122].

### 4.5. Ceramide 22

C22:0 Cer, composed of behenic (C22:0) or erucic (C22:1) acid, is primarily synthesized by CerS2 and CerS4 [21,26]. Although classified as a VLCCs, traditionally considered neutral or protective, emerging evidence links C22:0 Cer to adverse cardiometabolic phenotypes [157]. Key experimental and clinical studies are summarized in Table 7.

Preclinical studies demonstrate that CerS2 activity is essential for maintaining ceramide balance and ceramide and metabolic homeostasis. Raichur et al. [131] showed that CerS2 haploinsufficient in mice results in a profound depletion (90%) of VLCCs (C22:0–C24:0 Cer) and a compensatory accumulation (3.5-fold) of C16:0 Cer (*p* < 0.001). This shift was associated with impaired glucose tolerance, hepatic insulin resistance and steatohepatitis under a high-fat diet. Complete hepatic deletion of *CerS2* further impaired mitochondrial β-oxidation, as shown by reduced fatty acid oxidation rates, diminished respiratory capacity, and decreased activity of ETC complexes II and IV, leading to hepatic TAG accumulation (>2.5-fold versus controls).

Complementarily, Schmidt et al. [158] confirmed that *CerS2* silencing in hepatocytes reduced VLCCs (C22:0–C24:0 Cer) by ~65% (*p* < 0.001) and increased C16:0 Cer by ~48% (*p* < 0.01). These findings support that *CerS2* deficiency shifts dhSph metabolism towards CerS5/6, promoting the overproduction of C16:0 Cer, which are known to induce lipotoxicity, ER stress, and insulin resistance [131]. Thus, C22:0 Cer acts more as a buffer of LCCs than as an intrinsically toxic agent [160]. Maintaining CerS2 activity is crucial for preserving mitochondrial integrity and metabolic function, and its loss represents a tipping point towards adverse cardiometabolic phenotypes [131,160]. Mechanistically, C22:0 Cer modulates mitochondrial dynamics through its binding to the mitochondrial fission factor (MFF), which is essential for recruiting the GTPase Drp1 and promoting mitochondrial fragmentation.

Beyond its buffering role, Chen et al. [159] demonstrated that intestinal dysbiosis induced by *Phocaeicola vulgatus* interferes with the response to metformin in T2DM through hepatic accumulation of C22:0 Cer. In a prospective clinical cohort of drug-naïve patients with T2DM treated with metformin monotherapy (*n* = 162), non-responders exhibited a fecal abundance of *P. vulgatus* ~12-fold higher than responders (*p* < 0.001). In colonized murine models, hepatic C22:0 Cer levels increased 4.3-fold compared with controls (*p* < 0.01), accompanied by a 38.6% reduction in oxygen consumption rate (*p* < 0.01) and a significant decrease in mitochondrial membrane potential (*p* < 0.05).

Surface plasmon resonance assays confirmed that C22:0 Cer binds with high affinity to MFF, promoting Drp1 recruitment and mitochondrial fragmentation (+65% ± 8, *p* < 0.001). Pharmacological inhibition of fission with mdivi-1 partially reversed these effects, reducing fragmentation by ~40% (*p* < 0.01). Additionally, colonized animals displayed metabolic impairment, with HOMA-IR increased by ~52% (*p* < 0.01) and a 48% reduction in the hypoglycemic efficacy of metformin (*p* < 0.01). Collectively, these findings establish C22:0 Cer as a mechanistic link between the gut microbiota and hepatic mitochondrial function, driving mitochondrial fragmentation, OXPHOS dysfunction, and insulin resistance [159].

Human genetic and epidemiological data reinforce the clinical relevance of C22:0 Cer. Nicholson et al. [160] identified the *CerS2* rs267738 variant associated with lower hepatic expression of CerS2 and a ~28% reduction in C22:0 and C24:0 Cer (*p* < 0.01), correlating with increased insulin resistance and a higher incidence of coronary disease in the Utah CAD Study cohort (OR = 1.42; 95% CI, 1.10–1.83; *p* = 0.008).

Prospective evidence from the EPIC-Potsdam study (Wittenbecher et al. [161]) showed that elevated plasma levels of C22:0 Cer were associated with a higher risk of T2DM (HR = 2.77; 95% CI, 1.72–4.47; *p* < 0.001), even after adjustment for other Cer and metabolic risk factors. Through Mendelian randomization, the rs680379 variant in *SPTLC3* was identified as a genetic determinant of higher C22:0 Cer concentrations, confirming a causal relationship with diabetes risk. Dietary habits also play a role: high red meat consumption was associated with increased C22:0 Cer, while coffee consumption showed an inverse relationship, suggesting modulation of cardiometabolic risk via this Cer [161].

Finally, Zatloukal et al. [146] in the SHIP-START-1 cohort (*n* > 1100) evaluated the association between plasma ceramides and cardiorespiratory fitness. C22:0 Cer showed an inverse association with the maximum power achieved in exercise testing, adjusted for body weight in women under 54 years, with a significant sex-age interaction (*p* < 0.003). This finding suggests that, although it does not function as a marker of acute atherothrombotic events, C22:0 Cer may reflect a subclinical state of reduced functional capacity and cardiovascular vulnerability in specific subgroups.

Collectively, these findings indicate that C22:0 Cer is not a neutral lipid species but a context-dependent modulator of cardiometabolic health. Its effects are shaped by genetics, mitochondrial function, gut microbiota, and diet, supporting its potential role as biomarker and mediator of cardiometabolic risk.

### 4.6. Ceramide 24

C24:0 Cer is a very long-chain ceramide derived from lignoceric (C24:0) or nervonic (C24:1) acid [21,124] and is primarily synthesized by CerS2, with a contributory role reported for CerS3 in specific tissues [26,162]. The activity of CerS2 and the generation of VLCCs have been associated with cellular homeostasis and structural stability, particularly, in cardiac tissue. Key experimental and clinical studies are summarized in Table 8.

Preclinical evidence supports the protective role of CerS2-derived VLCCs at the cellular level. In human cardiomyocytes, Wiley et al. [163] demonstrated that CerS2 silencing exacerbates hypertrophic signaling and induced widespread transcriptional remodeling, including pathways related to lipid metabolism (SREBP), extracellular matrix organization, and pro-inflammatory signaling of the interferon-IL-4 axis (adjusted *p* < 0.05) [163]. Complementarily studies showed that co-expression of CerS2 with CerS4 or CerS6 enhanced VLCC synthesis and mitigated apoptosis and cytotoxicity induced by LCC-producing enzymes. [164]. Collectively, these findings support the sphingolipid rheostat concept, in which the balance between LCCs and VLCCs, rather than absolute ceramide levels, governs cell survival and stress responses.

However, the biological role of C24:0 Cer appears to be strongly context dependent. In metabolic stress models, Camacho et al. [165] reported increased plasma C24:0 Cer in rat pups on high-calorie diets, as well as in humans with obesity and T2DM (*p* < 0.001–0.0001). In experimental models, excess C24:0 Cer impaired glucose homeostasis, disrupted adipocyte differentiation, and promoted hepatic steatosis through altered mitochondrial fatty acid oxidation and activation of ER stress responses via SREBP1 and JNK. These findings indicate that while C24:0 Cer may exert protective structural functions in the myocardium, its systemic accumulation can contribute to metabolic dysfunction under conditions of nutrient excess [164].

Corroborating its role in inflammation-driven vascular pathology, levels of ceramide C24:1 Cer have been identified as elevated in populations of patients with high cardiovascular risk. In the cross-sectional study by Patyna et al. [166], patients with systemic lupus erythematosus (SLE) were investigated. The authors reported that a panel of LCCs, including C16:0, C18:0, C20:0, and C24:1 Cer, was significantly elevated in the plasma of patients with lupus nephritis (LN) compared to both healthy controls and SLE patients without renal involvement. C24:1 Cer was highlighted as the biomarker with the greatest potential, showing a particularly strong elevation in patients with lupus nephritis (*p* = 0.0001). Its association with the LN group strongly links elevated C24:1 Cer with a systemic proinflammatory state and active organ pathology, which provides supporting evidence for its role in inflammation-mediated vascular dysfunction.

Clinical data on C24:0 Cer as a cardiovascular biomarker are heterogeneous but informative. In the PREDIMED trial, higher plasma C24:0 Cer was associated with an increased risk of MACE (HR = 1.97; 95% CI: 1.21–3.20; *p*-trend = 0.004) [145]. In contrast, multiple cohorts of patients with stable CAD demonstrated neutral or protective associations, with lower C24:0 Cer levels observed in individuals who experienced cardiovascular death [44]. These observations are consistent with studies showing that higher C24:0 Cer is associated with lower CERT1 and CERT2 scores, reflecting a more favorable ceramide profile and minor cardiovascular risk [14,20,47,141].

In specific populations, C24:0 Cer appears to confer a protective signal. In individuals with long-standing T1DM, higher C24:0 was inversely associated with all-cause mortality (HR = 0.47; 95% CI: 0.31–0.73), representing one of the strongest protective associations reported for any ceramide species [151]. Similarly, higher concentrations of C24:0 Cer were observed in African American individuals without metabolic syndrome (*p* < 0.05), suggesting population-specific protective effects [167]. Together, these findings support the interpretation of C24:0 Cer as a modulatory or compensatory lipid species rather than a primary driver of vascular injury.

Although C24:0 Cer is present within atherosclerotic plaque, its direct pathogenic contribution remains less clearly defined than that of LCCs such as C16:0 Cer [143,152]. Current evidence suggests that C24:0 Cer participates in the broader lipotoxic milieu rather that acting as an independent proinflammatory mediator. Further mechanistic studies are required to determine whether C24:0 Cer exert context-specific protective or modulatory effects within vascular tissue [152,168]. Clinically, these data support the use of C24:0 Cer primarily in ratio-based indices, where they counterbalance lipotoxic ceramides and improve cardiovascular risk stratification.

## 5. Therapeutic Strategies for the Modulation of Ceramide Metabolism

### 5.1. Current Lipid-Lowering Drug Therapies

Statins, in addition to inhibiting HMG-CoA reductase and reducing cholesterol synthesis, also significantly modulate sphingolipid metabolism [169,170]. SREBP-2 is the principal regulator of genes involved in cholesterol synthesis and the LDL receptor (LDLR) [155]. By inhibiting HMG-CoA reductase, statins lower ER cholesterol levels, thereby activating the processing and nuclear translocation of SREBP-2 [155]. Unlike their effects on LDL-C, statins do not directly inhibit ceramide synthesis; instead, they exert an indirect regulatory effect on circulating Cer levels [135].

Cer can impair the maturation of transcriptionally active forms of SREBP-1 and SREBP-2, suggesting that their pharmacological reduction may partially relieve this inhibitory effect. Furthermore, SREBP-2 activation influences broader lipid metabolic pathways, indirectly modulating enzymes within phospholipid and sphingolipid biosynthetic networks [171]. Sterol depletion alters ER lipid composition and can modify SPT activity, indicating that SPT-dependent lipid homeostasis is required for an appropriate SREBP-mediated response to cholesterol deficiency [172]. Lipidomic studies have consistently shown that statins reduce multiple Cer species, although the magnitude of this response varies substantially between individuals.

In patients with metastatic prostate cancer treated with simvastatin as a metabolic intervention to correct an unfavorable lipid signature, Mak et al. [173] reported marked reductions in ceramides, hexosylceramides, and sphingomyelins, with decreases ranging from −23% to −52% (*p* < 0.05). Among the most affected species were Cer 24:0, HexCer 16:0, and SM 18:1/16:0, which declined by −28% to −46%. However, only 45% of patients achieved lipid signature normalization, corresponding to a mean shift of −2.35 SD, compared with an expected 19% spontaneous normalization rate, revealing a subgroup with incomplete reductions (<20–25%) despite adequate decreases in LDL-C.

Similarly, in individuals with MS, Ng et al. [170] identified a clear dose–response relationship with rosuvastatin: ceramides such as Cer 16:0 decreased by −31% with 10 mg and −36% with 40 mg, and Cer 24:1 declined by −29% vs. −34%. Sphingomyelins decreased by −27% vs. −31%, whereas LDL-C fell by −38% vs. −49%, representing the most pronounced reduction among lipid classes. Cer also showed very strong correlations with apolipoprotein C-III (apoC-III): baseline r = 0.80 (*p* = 0.002), attenuating yet remaining strong with high-dose therapy (r = 0.69, *p* < 0.01). After adjustment, partial correlations persisted at high levels (r = 0.59–0.81), supporting continued hepatic Cer production that is only partially sensitive to statin-driven lipoprotein clearance.

Complementarily, Bergheanu et al. [174] demonstrated that rosuvastatin produced a more pronounced reduction in the sphingomyelin-to-phosphatidylcholine ratio (SPM/(SPM+PC), with an approximate decline of −18% (*p* ≤ 0.05), compared with atorvastatin (−10%). In contrast, atorvastatin reduced sphingomyelins more strongly at higher doses (−22% to −28%). Both statins also lowered VLCCs including C22:0, C24:0, and C24:1 Cer by approximately −15% to −35%, as quantified by LC–MS.

Ezetimibe, a selective inhibitor of the Niemann–Pick C1-Like 1 (NPC1L1) protein, is the most widely used second-line therapy for lowering LDL-C [175,176]. It acts at the brush border of enterocytes, blocking the absorption of dietary and biliary cholesterol. Although NPC1L1 has been classically described as a sterol transporter, emerging evidence suggests an additional role in sphingolipid absorption [176]. Experimental data indicate that NPC1L1 facilitates intestinal uptake of SM and its metabolites.

Yamanashi et al. [176] showed in murine models that NPC1L1 functions as a physiological importer of SM, regulating its conversion to S1P and its incorporation into VLDL-C and LDL-C particles. Using in vivo absorption assays with radiolabelled ^3^H-SM and LC–MS, inhibition of NPC1L1 with ezetimibe reduced intestinal SM absorption by more than 50% and attenuated apoM-associated S1P production in atherogenic lipoproteins, suggesting a mechanism through which dietary ceramide availability may be diminished. In contrast to these mechanistic findings, clinical evidence indicates that ezetimibe has a neutral effect on circulating ceramides.

In the LURIC cohort, Tarasov et al. [155] compared Simvastatin, Ezetimibe, and their combination using LC–MS and shotgun lipidomics. Simvastatin reduced ceramides and cerebrosides by approximately 25%, accompanied by a 40% reduction in LDL-C. By comparison, Ezetimibe monotherapy, despite a 21% decrease in LDL-C, did not significantly modify plasma ceramide concentrations; some species, including C18:0 Cer and C20:0 Cer, increased modestly (+7.6% and +8.8%). Further evidence in patients with MI treated with Rosuvastatin or Simvastatin plus Ezetimibe using high-resolution lipidomics (Lipidyzer) [175], showed that Rosuvastatin reduced Cer by 37% and SM by 36% after 30 days, whereas the Simvastatin–Ezetimibe combination produced minimal changes in Cer and only modest reductions in SM (−27%). Correlations between Cer concentrations and cardiovascular risk persisted only in the rosuvastatin group, suggesting that ezetimibe confers no incremental benefit on Cer modulation beyond its LDL-C-lowering effect.

The use of PCSK9 inhibitors such as alirocumab and Evolocumab, as well as the siRNA, inclisiran, has been established in patients with high cardiovascular risk or familial hypercholesterolemia, demonstrating efficacy in lowering LDL-C and reducing cardiovascular events [177]. Proprotein convertase subtilisin/kexin type 9 (PCSK9) is a serine protease secreted primarily by the liver that binds to the extracellular EGF-A domain of the LDLR [178]. Upon internalization of the PCSK9–LDLR complex, PCSK9 affinity increases in the acidic environment of the endosome, preventing receptor dissociation and recycling [178,179]. Consequently, the LDLR is directed to lysosomes for degradation rather than being recycled to the cell surface [178]. Inhibition of PCSK9 prevents this degradation, enhances LDLR expression on the hepatocyte membrane, and reduces plasma LDL-C concentrations [177].

CD36 is an integral transmembrane protein that facilitates the uptake of long-chain fatty acids and oxidized lipoproteins in adipocytes, hepatocytes, and macrophages [178,179]. PCSK9 promotes CD36 degradation in adipocytes and hepatocytes, thereby limiting fatty acid uptake and TAG accumulation in tissues such as the liver and visceral adipose tissue [178]. Blocking the interaction between PCSK9 and CD36 stabilizes CD36 on the plasma membrane [178], increasing the flux of long-chain fatty acids into cells and enhancing lipid storage in peripheral and hepatic tissues. This process results in a significant decrease in circulating bioactive lipids, including ceramides and cholesteryl esters [180,181]. It has been demonstrated that carriers of the R46L loss-of-function variant in PCSK9, exhibited a sustained reduction in plasma ceramides associated with fatal CAD outcomes [155]. These reductions reached approximately 20%, particularly in species such as C16:0 Cer and C18:0 Cer, conferring cardiovascular protection. Moreover, ceramide ratios confirmed their predictive value: Cer(16:0/24:0) with an adjusted OR of 1.62 and Cer(20:0/24:0) with an adjusted OR of 1.50 were associated with increased risk (*p* < 0.05), whereas Cer(24:0/24:1) demonstrated a protective effect (adjusted OR 0.65, *p* = 0.0003).

In patients with familial hypercholesterolemia treated with Evolocumab for 12 weeks, there was a reduction in VLCCs, together with hexosylceramides and lactosylceramides, with robust associations (*p* < 0.05 after FDR correction) [181]. Additionally, decreases in the unsaturation indices of these species were observed, indicating qualitative remodeling of circulating sphingolipids. Finally, Hilvo et al. [180] analyzed 40 patients with CAD enrolled in the EQUATOR clinical trial, in which the monoclonal antibody RG7652 was administered to inhibit PCSK9. Using a detailed lipidomic approach, the authors demonstrated that PCSK9 inhibition significantly reduced ceramides, dihydroceramides, and sphingomyelins, with the most pronounced effects observed in species containing very long-chain fatty acids (22:0, 24:0, and 26:0). These reductions reached magnitudes of 37–44% (*p* < 0.01–0.001).

### 5.2. Pharmacological Therapies That Modulate Ceramide Production or Degradation

Table 9 summarize the strategies modulating ceramide metabolism and their associated physiological effects.

Fumonisin B1 (FB1), a mycotoxin produced by species of the genus *Fusarium*, was reported more than three decades ago as an inhibitor of ceramide synthesis [192]. Its toxicity is attributed to its structural similarity to natural sphingoid bases, primarily dhSph and Sph [182]. Its main molecular target is CerS. FB1 acts as a competitive inhibitor of CerS substrates (dhSph and fatty acyl-CoA); its sphingoid base-like region mimics dhSph or Sph, while its tricarballylic acid (TCA) side chains interfere with acyl-CoA binding and prevent normal ceramide formation [192].

The inhibition of CerS by FB1 causes a profound disruption of sphingolipid metabolism, which is considered the immediate cause of its toxicity [182,192]. This blockade generates a systemic lipid imbalance, with a reduction in Cer, dhCer, and complex sphingolipids, and an accumulation of dhSph, Sph, and their phosphorylated forms, due to the fact that SPT remains active or even increases its activity [192]. These events provoke a lipid storm that disrupts cellular homeostasis, compromises membrane integrity, and perturbs signaling pathways related to proliferation, apoptosis, and stress response, explaining the hepatotoxic, nephrotoxic, and carcinogenic effects of FB1 in animal and human models [182,192].

In in vitro studies with rat hepatocytes, Wang et al. [192] showed that FB1 competitively inhibits CerS with an IC_50_ of ~0.1 μM, reducing Cer synthesis by 50%. At 1 μM, inhibition was nearly complete (~90–100%), and dhSph levels increased ~110-fold after four days of exposure. In vivo, hepatotoxicity and nephrotoxicity have been demonstrated in animals; for example, calves exposed to 1 mg/kg/day developed liver lesions and proximal tubular dysfunction [193]. Co-exposure to FB1 and aflatoxin B1 has been proposed as a synergistic factor in hepatic and esophageal carcinogenesis, though human evidence remains largely ecological [194].

Kurek et al. [183] evaluated myriocin, a fungal metabolite and potent SPT inhibitor, in rats with HFD-induced insulin resistance. Myriocin significantly improved systemic insulin sensitivity (*p* < 0.05), reducing HOMA-IR by ~67% (from 20.0 ± 2.5 to 6.6 ± 1.3). Cer, dhSph, and intramuscular TAG were also significantly reduced (*p* < 0.05), demonstrating an insulin-sensitizing effect. Morrice et al. [184] tested fenretinide (30 mg/kg/day for 10 weeks) in obese, insulin-resistant mice. Treatment improved glucose tolerance, reducing AUC by ~40% (*p* < 0.01), and enhanced insulin sensitivity (*p* < 0.05). Hepatic ceramides decreased by ~35% (*p* < 0.01), while plasma FGF21 levels fell by ~55% (from ~420 to ~190 pg/mL, *p* < 0.001). At the molecular level, fenretinide downregulated Sptlc1 and Degs1 and modulated retinoid and metabolic genes in liver and adipose tissue.

ACDase, encoded by ASAH1, hydrolyzes Cer into Sph and fatty acids. Upregulation reduces pro-apoptotic ceramides and promotes survival in cancer [185]. Draper et al. [185] identified Ceranib-2 as a potent ASAH1 inhibitor (IC_50_ = 0.71 ± 0.08 μM for purified enzyme; 1.3 ± 0.2 μM in SKOV3 cells). Ceranib-2 increased intracellular ceramides ~2.5-fold (*p* < 0.01), reduced Sph by 60% and S1P by 55%, and shifted the sphingolipid rheostat toward apoptosis. Combined with paclitaxel, Ceranib-2 reduced cell growth by ~70% (*p* < 0.001).

Chen et al. [186] developed novel ceramide analogs with therapeutic potential against virus-associated primary effusion lymphoma (PEL). These compounds increased intracellular ceramides up to 2.5-fold, upregulated CerS1/3/4 expression 2–3-fold, and downregulated SphK1/2 and GCS by 40–60%. This dual modulation induced apoptosis in >50% of PEL cells, demonstrating that ceramide analogs not only act as lipid mimetics but also reprogram sphingolipid metabolism toward a pro-apoptotic state.

Recent studies have identified Nogo-A as an inducible regulator of ceramide biosynthesis in the heart, acting through direct inhibition of SPT. Unlike its well-established role in the central nervous system as an inhibitor of neurite outgrowth, Nogo-A in cardiomyocytes functions as a metabolic safeguard, transiently upregulated in response to hemodynamic stress such as transverse aortic constriction (TAC) or chronic hypertension [188,189]. This upregulation is not pathological but reflects a homeostatic adaptation to increased biosynthetic demand. By binding to the catalytic subunit SPTLC1 at the endoplasmic reticulum membrane, Nogo-A imposes an allosteric constraint that reduces SPT’s maximal catalytic velocity (Vmax) without altering substrate affinity (Km), thereby limiting ceramide accumulation and preventing lipotoxicity [195].

Although both Nogo-A and Nogo-B can inhibit SPT, their compartmentalized expression defines distinct physiological roles: Nogo-B predominates in endothelial cells, modulating vascular tone and blood pressure via S1P signaling [196], whereas Nogo-A is selectively induced in cardiomyocytes to preserve mitochondrial integrity and contractile function by restraining ceramide synthesis [188]. The cardioprotective role of Nogo-A has been validated in loss-of-function models. Mice with cardiomyocyte-specific deletion of Nogo-A (Nogo-A CM-KO) subjected to TAC exhibited a 50% mortality rate at 3 months, compared to <15% in wild-type controls. Kaplan–Meier survival curves revealed a statistically significant divergence (*p* < 0.01, Log-rank test), underscoring Nogo-A as a determinant of survival under sustained mechanical stress [188].

#### Selective Inhibition of CerS Isoforms

The therapeutic interest in Cer has driven the development of strategies aimed at selectively reducing bioactive species most associated with metabolic dysfunction, particularly C16:0 Cer, while preserving variants essential for cellular integrity. Among the most studied approaches are gene inhibition using antisense oligonucleotides (ASOs) and small-molecule inhibitors derived from Fingolimod (FTY720), a Sph analog with immunomodulatory properties.

Raichur et al. [131] demonstrated that haploinsufficiency of CerS2—the dominant hepatic isoform synthesizing VLCCs (C22:0–C24:1)—increases susceptibility to high-fat diet induced steatohepatitis and insulin resistance. Although total Cer levels remained unchanged, CerS2^+^/^−^ mice exhibited a compensatory rise in C16:0 Cer (*p* < 0.0001), a ~40-fold upregulation of FSP27 (*p* < 0.001), reduced activity of ETC complexes II and IV (*p* < 0.001), and a ~30% decrease in fatty acid oxidation (*p* < 0.05). These changes promoted hepatic TAG accumulation, elevated ALT/AST, and impaired Akt signaling, confirming an adverse metabolic phenotype.

In contrast, Turpin et al. [130] identified CerS6 and its main product, C16:0 Cer, as key mediators of obesity-associated metabolic dysfunction. In humans, CERS6 expression in visceral adipose tissue correlated positively with BMI (r = 0.42, *p* < 0.001) and negatively with insulin sensitivity (r = −0.38, *p* < 0.01). Consistently, CerS6^−^/^−^ mice fed a HFD showed a ~45% reduction in C16:0 Cer across liver, muscle, and adipose tissue (*p* < 0.01), accompanied by lower body weight (−25%, *p* < 0.05), improved glucose tolerance (AUC reduced by ~40%, *p* < 0.01), and protection against insulin resistance. These effects were associated with increased energy expenditure (+20%, *p* < 0.05) and restoration of hepatic Akt phosphorylation. Liver-specific deletion (CerS6^ΔLIVER^) reproduced these benefits, confirming the central role of CerS6 in metabolic homeostasis.

Schiffmann et al. [187] developed FTY720 analogs with isoform-specific inhibition profiles. ST1060 preferentially inhibited CerS2 (IC_50_ ≈ 63 μM in vitro), whereas ST1072 was more potent against CerS4 and CerS6 (IC_50_ = 50.4 μM for C16:0; 26.9 μM for C18:0; cellular inhibition of C16:0 with IC_50_ = 6.8 μM, *p* < 0.01). ST1058 and ST1074 showed selectivity toward CerS2/4. These findings demonstrate the feasibility of chemical inhibition targeting specific isoforms and open the possibility of designing selective compounds against CerS5/6, which are implicated in obesity, insulin resistance, and CVD. Beyond direct inhibition, post-translational regulation of CerS represents an emerging therapeutic mechanism.

Sassa et al. [71] showed that CerS2–CerS6 isoforms possess multiple phosphorylation sites at their C-terminal ends, modulating catalytic activity and acyl-CoA specificity. Dephosphorylation of CerS5/6 reduced enzymatic activity by 40–60% (*p* < 0.01). Casein kinase 2 (CK2) was identified as a key regulatory kinase, and its pharmacological inhibition with CX-4945 (Silmitasertib) decreased CerS phosphorylation and reduced total Cer levels in cell cultures by ~35% (*p* < 0.05). This suggests that reversible modulation of CerS5/6 via CK2 may offer a safer therapeutic window than direct inhibition. Overall, the evidence indicates that selective inhibition of CerS isoforms or pharmacological modulation of their activity arises as a promising strategy for the treatment of metabolic and CVD.

### 5.3. Non-Pharmacological Therapies

Non-pharmacological interventions, particularly dietary modification and physical exercise, constitute relevant strategies for modulating the availability of substrates destined for ceramide synthesis and, consequently, their circulating profile [190,197].

#### 5.3.1. Dietary Evidence

Diet plays a decisive role by providing the fatty acids that serve as precursors for the endogenous formation of ceramides. Diets rich in saturated fatty acids are associated with an increase in plasma ceramide levels, which is linked to the development of hepatic steatosis and insulin resistance. In contrast, the intake of polyunsaturated fatty acids (PUFA) has been associated with a reduction in serum Cer concentrations. Moreover, the restriction of dairy products and the supplementation with fibers and polyphenols have shown beneficial effects in modulating circulating levels of these lipid species [190,198]. The PREDIMED clinical trial serves as a benchmark in this field.

Wang et al. [145] analyzed 980 participants (230 incident CVD cases) over a median follow-up of 7.4 years. Plasma ceramides C16:0, C22:0, C24:0, and C24:1 was positively associated with cardiovascular incidence, with adjusted relative risks ranging from 1.73 to 2.39 when comparing extreme quartiles (*p* < 0.05). A composite ceramide score was associated with a more than twofold increase in CVD risk (HR = 2.18; 95% CI: 1.36–3.49; *p* < 0.001).

Importantly, adherence to the Mediterranean diet (MedDiet) significantly attenuated this association (interaction *p* = 0.010), with a stronger protective effect in the extra virgin olive oil-supplemented group (*p* = 0.012) compared with the nut-supplemented group (*p* = 0.037). Notably, total ceramide concentrations did not change significantly after one year of intervention, suggesting that the benefits of the MedDiet are mediated not by a quantitative reduction in ceramides but by qualitative shifts in species composition (e.g., lower C16:0 Cer and higher C24:0 Cer) and by the anti-inflammatory and antioxidant properties of PUFAs and polyphenols [145].

An unexpected finding emerged from the FRUVEDomic study (Mathews et al. [199]), an 8-week intervention in 50 young adults based on the Dietary Guidelines for Americans and emphasizing fruit and vegetable intake. The intervention improved metabolic parameters (waist circumference, systolic blood pressure, and total cholesterol) and significantly reduced plasma ceramide C24:0 Cer (~50% reduction, *p* < 0.01). However, ceramide C16:0 Cer increased by ~50–80% (*p* < 0.05), resulting in a ~66% increase in the C16:0/C24:0 Cer ratio. This shift suggests that while dietary interventions may improve overall metabolic health and reduce certain ceramide species, they can simultaneously promote a relative enrichment of more bioactive and potentially pathogenic ceramides.

#### 5.3.2. Evidence of Physical Exercise

Exercise constitutes another widely validated non-pharmacological intervention to improve insulin sensitivity, partly through the modulation of ceramide metabolism in skeletal muscle [197]. Chronic training stimulates the uptake and oxidation of fatty acids (β-oxidation) in muscle fibers, diverting lipid substrates away from the de novo Cer synthesis pathway and thereby reducing their intracellular accumulation [197].

Helge et al. [191] demonstrated that both acute and prolonged exercise modify Cer metabolism in humans, regardless of training status. These effects include changes in Cer species content and in Mg^2+^-dependent nSMase activity, suggesting a dual mechanism: reduction of de novo synthesis by substrate diversion and promotion of the catabolism of preexisting Cer. In trained and untrained healthy subjects, muscle biopsies were performed before and after a prolonged exercise session (3 h at 58% of VO_2_max). The results demonstrated a 25% increase in total Cer fatty acid content in both groups, accompanied by a significant decrease in nSMase activity (*p* < 0.05).

These findings confirm acute and sustained exercise directly impacts ceramide synthesis and degradation dynamics in human skeletal muscle, contributing to improved metabolic homeostasis and insulin sensitivity

## 6. Perspectives, Limitations and Future Directions

In the next decade, clinical research on ceramides is expected to expand significantly, driven by advances in high-resolution omics platforms and data-science–based analytical approaches. A major shift will likely move the field from linear or reductionist interpretations toward an integrated understanding of how ceramide species participate in complex metabolic networks. Risk stratification may benefit from pan-omics approaches that combine genomic, transcriptomic, lipidomic, and microbiome data using advanced computational tools to refine metabolic subphenotypes and improve disease-trajectory modeling.

Future research should prioritize standardized ceramide measurement, longitudinal validation across diverse populations, and interventional studies targeting ceramide metabolism. Integrating ceramide profiling with multi-omic approaches may further enhance cardiovascular risk prediction and therapeutic precision.

Research may extend beyond cardiometabolic disorders. Areas such as the liver–brain axis or metabolic oncology could elucidate the contribution of ceramides to cerebral insulin resistance, cognitive decline, or tumor sensitization to antineoplastic therapies.

A major challenge will be the translation of ceramide quantification from specialized laboratories to routine care. Emerging point-of-care technologies based on microfluidics or electrochemical sensing may facilitate rapid and cost-effective measurement of clinically relevant ceramide panels. Although standardization barriers remain, current progress suggests that the integration of ceramides into precision-medicine frameworks will become increasingly feasible as longitudinal and rigorously designed clinical studies accumulate.

This review has several limitations. First, its narrative design precludes quantitative synthesis and formal risk-of-bias assessment. Second, heterogeneity in lipidomic platforms and reporting standards limit direct comparison across studies. Third, most clinical evidence remains observational, preventing causal inference.

## 7. Conclusions

Ceramides have emerged as key bioactive lipids with important roles in cardiovascular and metabolic pathophysiology. Distinct species: from LCCs such as C14:0 Cer and C16:0 Cer to very VLCCs like C22:0 Cer and C24:0 Cer, exert divergent biological effects, ranging from promoting lipotoxicity, inflammation, and apoptosis to contributing to mitochondrial regulation and cellular energy homeostasis. Clinically, plasma ceramide profiles have shown consistent diagnostic and prognostic value, with C16:0 Cer and C18:0 Cer repeatedly associated with higher cardiovascular risk and mortality, whereas VLCCs appear to reflect compensatory or protective states. Their incorporation into lipidomic panels and indices such as CERT1 and CERT2 has improved prediction of MACE beyond traditional lipid markers.

Mechanistic and translational studies have demonstrated their relevance as potential therapeutic targets, supported by experimental evidence on CerS inhibition, ceramidase activation, and lifestyle interventions capable of reducing harmful ceramide species. However, the field remains limited by methodological heterogeneity in lipidomic quantification, a paucity of large clinical studies, and the absence of standardized clinical thresholds. The current evidence positions ceramides as integrative biomarkers that reflect metabolic injury and organ-level dysfunction, with meaningful implications for early diagnosis, risk stratification, and mechanistic understanding of cardiovascular and metabolic diseases. Their growing incorporation into clinical and translational research highlights their relevance within contemporary precision-medicine paradigms

## Figures and Tables

**Table 1:** Ceramide synthases: acyl-CoA chain length specificity and tissue distribution.

CerS.	Preferential Acyl-CoA Substrate	Tissue Expression
CerS1	18 carbons	Brain, skeletal muscle and testis
CerS2 *	20–26 carbons	Nervous system (oligodendrocytes, Schwann cells), kidney and liver
CerS3	>26 carbons	Testicle and skin (keratinocytes)
CerS4	18–20 carbons	Most tissues, predominantly in leukocytes, liver, heart and skin
CerS5	16 carbons	Most tissues. Higher expression in the pulmonary epithelium
CerS6	14 and 16 carbons	Most tissues. Predominantly in the intestine and kidney

*: CerS more widely expressed. CerS: ceramide synthases. Data obtained of [26,62,63,64].

**Table 2:** Foundational and validation studies in the development of ceramide-based cardiovascular risk scores.

Author	Ceramides Measured	Type of Study, Population, Details	Findings
Havulinna (2016) [19]	Ceramides (d18:1/16:0, 18:0, 24:0, 24:1) and analysis of ratios of 16, 18 and 24:1 carbon ceramide versus 24:0 carbon ceramide.	Observational-prospective nested within a cohort; (*n*): 8101; Apparently healthy Finnish subjects. Pioneer in the creation of the CERT1 score Validated ceramides in the general population, not just in patients at high cardiovascular risk.	Relative risk increase of 2.7-fold for MACE and 4.3-fold for death due to MACE. The Cer(d18:1/18:0) species showed the most significant individual association, presenting a Hazard ratio (HR) of 1.21 (95% CI: 1.11–1.33) for incident MACE after adjustment for Framingham risk factors. The combination with C-reactive protein is proposed to identify very high-risk subjects.
Meeusen (2018) [117]	Ceramides of 16, 18, 24, and 24:1 carbon were analyzed individually, as well as their ratios to the 24:0 carbon ceramide.	Prospective cohort of US patients referred for coronary angiography (*n*): 504. Indirectly describes the development of the CERT1 score, subsequently the MAYO clinic developed the commercial version in collaboration with Zora Biosciences Oy under the leadership of Hilvo and Laaksonen.	Patients in the highest risk category (Score ≥ 10) had a more than 2-fold higher risk of 4-year MACE and 18-year mortality compared with the low-risk group. The Ceramide Risk Score (16:0)/(24:0) ratio was the most potent predictor, with an adjusted Hazard Ratio of 1.75 per SD for MACE. The prognostic utility of these molecules was further consolidated by the Ceramide Risk Score (adjusted HR of 1.58 per SD).
Hilvo (2020) [110]	Individually, ceramides with 16, 18, 24, and 24:1 carbon atom were analyzed, as well as their ratios relative to the 24:0 carbon ceramide, and the measurement of phosphatidylcholines (PC 16:0/22:5, PC 14:0/22:6, and PC 16:0/16:0) was also added.	The CERT2 score was developed based on three studies; the score was developed in the WECAC cohort and validated in the LIPID and KAROLA cohorts, based on its use in secondary prevention for patients with established atherosclerotic coronary artery disease.	CERT2 proved to be a highly significant predictor of cardiovascular death, surpassing traditional lipids such as LDL-C. Its combination with troponin T (CERT2-TnT) drastically improved residual risk stratification, allowing for the precise identification of 10–20% of patients at extremely high risk.
Hilvo (2021) [112]	The same ones included in the CERT2 score	Based on the FINRISK 2002 cohort, used to test its efficacy in primary prevention, excluding those patients with prevalent cardiovascular events.	CERT2 proved to be a significant and robust predictor of all cardiovascular outcomes, particularly for fatal events. It was demonstrated that the incidence of fatal events was more than 10 times higher in the highest CERT2 risk category compared to the lowest.
Wenjie Yin (2021) [118]	Seven different ceramide species were measured; however, the score only includes Cer (18:1/16:0) and Cer (18:1/24:1), along with their proportions relative to Cer (18:1/22:0) and Cer (18:1/24:0).	They developed the CERT-HBP score using a prospective analysis model within the PROSPECT study cohort of patients with essential hypertension who were classified in a high or very high cardiovascular risk category.	The CERT-HBP allows for enhanced identification of high-risk patients, whose highest category showed a 4.6-fold increase in the risk of MACE compared to the lowest category.

Abbreviations: MACE: major adverse cardiovascular events; HR: hazard ratio; SD: standard deviation; CI: confidence interval; PC: phosphatidylcholines; LDL-C: low-density lipoprotein cholesterol.

**Table 3:** Summary of C14:0 ceramide mechanisms across experimental and clinical context.

Study Type (Reference)	Biological Matrix	Organelle Involved	Observed Pathophysiological Mechanism
In vitro [120]	Human hepatocytes (HepG2)	Mitoc + RE (functional interaction)	MA ↑ C14:0-CoA incorporation → ↑ C14:0 Cer; in the presence of PA → synergistic effect → ↑ Total Cer ↓ SM → ER stress + mitochondrial dysfunction → cytochrome c ↑ → caspases ↑ → apoptosis Result: exacerbated lipotoxicity (MA potentiates PA-induced lipotoxicity)
In vitro [121]	Primary cardiomyocytes	Mitoc + RE	MA (C14:0) → CerS5 ↑ → ↑ C14:0 Cer → ↑ autophagy (↑ BECN1, ↑ LC3B, ↑ Atg7) → ER/mitocytic remodeling → cardiomyocyte hypertrophy
In vivo [121]	Mouse heart		Inhibition of CerS5 (siRNA) or de novo synthesis (myriocin) → ↓ C14:0 Cer → ↓ autophagy → ↓ hypertrophy
Clinical [13,122]	Plasma	Systemic	MI → ↑ Plasma C14:0 Cer → activates mitochondrial pathways (autophagy/apoptosis) → worse prognosis (↑ MACE)
[123]			Aerobic exercise → ↓ Plasma C14:0 Cer → negative correlation with ↑ insulin sensitivity (↓ Akt inhibition → ↑ glucose uptake)
In vivo [13]	Myocardium	Mitoc + RE	HF/MI patients→ ↑ SPTLC2 → ↑ de novo Cer (including C14:0) in plasma and myocardium → pathological remodeling (fibrosis, inflammation, apoptosis). SPT inhibition (myriocin) or SPTLC2 deletion → ↓ Cer → ↓ remodeling and improved function

MA: myristic acid; PA: palmitic acid; Cer: ceramide; SM: sphingomyelin; ER: endoplasmic reticulum; Mitoc: mitochondria; CerS5: ceramide synthase 5; SPT: serine palmitoyltransferase; SPTLC2: SPT subunit 2; MI: myocardial infarction; HF: heart failure; MACE: major adverse cardiovascular events; ↑: Increase; ↓: Decrease.

**Table 5:** Summary of C18:0 Ceramide Mechanisms Across Experimental and Clinical Context.

Study Type (Reference)	Biological Matrix	Organelle Involved	Observed Pathophysiological Mechanism
In vitro [148]	HUVEC	RE + Mitoc	Aldosterone → MR → CerS1 ↑ → C18:0 Cer ↑ (ER) ⟶ Accumulation C18:0 Cer → ↑ pro-apoptotic signaling ⟶ Mitochondria: dysfunction + amplification of apoptotic cascade ⟶ Caspase-3 activation → DNA fragmentation → endothelial apoptosis
			Modulators: Eplerenone (MR antagonist) ⟶ ↓ C18-Cer → protection S1P (anti-ceramide lipid) ⟶ ↓ apoptosis PDMP (GCS inhibitor) or C6:0 Cer exogenous ⟶ ↑ apoptosis *CerS1* shRNA ⟶ ↓ C18:0 Cer → protection *CerS1* upregulation ⟶ ↑ C18:0 Cer → ↑ apoptosis
In vitro	Macrophages (RAW264.7)	RE + Mitoc	TAD → ↑ C18:0 Cer (plasma, aortic macrophages) ⟶ activation of NLRP3 inflammasome ⟶ ↑ casp-1 act ⟶ ↑ IL-1β, IL-18 ⟶ ↑ MMPs (MMP9) ⟶ ECM degradation + aortic wall weakening ⟶ ↑ inflammation + ↑ risk of dissection
Murine BAPN-TAD model	In vivo		Exogenous C18:0 Cer ⟶ ↑ macrophage inflammation + ↑ MMPs
Clinical [149]	plasma metabolomics in TAD vs. TAA vs. controls		Myriocin (SPT inhibitor) ⟶ ↓ de novo Cer synthesis ⟶ ↓ aortic inflammation + ↓ TAD incidence in mice
Clinical [150]	Plasma (patients with MS)	RE	MS → ↑ plasma C18:0 Cer ⟶ linked to SNPs in ceramide biosynthetic genes (CERS3, CERS6, SGMS1, SPTLC2/3, ACER1) ⟶ dysregulated sphingolipid metabolism ⟶ ↑ insulin resistance (HOMA-IR ↑, insulin ↑) ⟶ ↓ adiponectin, ↑ TAG, ↑ waist circumference, ↑ BP ⟶ systemic metabolic stress and cardiovascular risk.
Clinical [44,145]	Plasma (patients with CAD, ACS, high CV risk)	Systemic	↑ Plasma C18:0 Cer ⟶ associated with ↑ CV death and ↑ incident CVD.
			Inferred mechanism: C18:0 Cer ↑ → lipoprotein uptake dysregulation + vascular inflammation + apoptosis (Mitoc stress) → atherosclerotic plaque instability → MI, stroke, CV death.
			Ratios (C18:0/C24:0 Cer) stronger predictors than absolute levels. Mediterranean diet (EVOO/nuts) attenuates risk despite high ceramide score.

Cer, ceramide; CerS, ceramide synthase; MR, mineralocorticoid receptor; SPT, serine palmitoyltransferase; LCC, long-chain ceramide; ECM, extracellular matrix; MMP, matrix metalloproteinase; HF, heart failure; MI, myocardial infarction; MACE, major adverse cardiovascular events; TAD, thoracic aortic dissection; TAA, thoracic aortic aneurysm; MS, metabolic syndrome; T1DM, type 1 diabetes mellitus; T2DM, type 2 diabetes mellitus; LDL-C, low-density lipoprotein cholesterol; ACS, acute coronary syndrome; ↑: increase; ↓: decrease.

**Table 6:** Summary of C20:0 Ceramide Mechanisms Across Experimental and Clinical Context.

Study Type (Reference)	Biological Matrix	Organelle Involved	Observed Pathophysiological Mechanism
Clinical	Plasma (AAD patients)	Systemic	Inferred mechanism: Intestinal FXR ↑ → Cers2 ↑ → C20:0 Cer ↑ (plasma) ⟶ macrophage inflammatory transformation ⟶ ↑ MMP2/MMP9 release ⟶ ECM degradation (↓ COL1, ↓ FN1) ⟶ vascular wall weakening ⟶ ↑ incidence of AAD
In vivo	Mouse (BAPN models)	ER + Mitoc	Myriocin (SPT inhibitor) ⟶ ↓ de novo synthesis → ↓ AAD
			Exogenous C20:0 Cer ⟶ directly ↑ MMP2/MMP9 from macrophages, fibroblasts, VSMCs.
In vitro [153]	Enterocytes and macrophages (BMDM)		UDCA (FXR inhibitor) ⟶ ↓ FXR activation → ↓ Cers2 → ↓ C20:0 Cer → ↓ inflammation + preserved ECM → ↓ AAD incidence
In vivo	Mouse MI model (LAD ligation)	ER + Mitoc + Lysosome	MI → hypoxia/ischemia → de novo Cer synthesis ↑ → C20:0 Cer ↑ (16.5-fold in LV, 24h post-MI) ⟶ MITOC stress + casp-3 dimerization/cleavage ⟶ cardiomyocyte apoptosis ↑ ⟶ LV dysfunction + scar formation
In vitro [154]	LV Cardiomyocytes (myocardium under hypoxia)		aCDase inhibition ⟶ ceramide accumulation ↑ → cardiomyocyte death ↑ → survival ↓/aCDase modRNA overexpression ⟶ C20:0 Cer ↓ → sphingosine ↑ → S1P ↑ (pro-survival) ⟶ ↓ apoptosis, ↓ neutrophil infiltration, ↓ inflammation ⟶ improved LV function + survival.
Clinical [155]	Plasma (CAD patients)	Systemic	↑ C20:0 Cer ⟶ associated with ↑ CV death risk in CAD Ratio C20:0/C24:0 Cer = strong predictor of fatal outcome, independent of LDL-C.
			Simvastatin ⟶ ↓ C20:0 Cer (~25%)/Ezetimibe ⟶ LDL-C ↓ but C20-Cer unchanged/slightly ↑/PCSK9 LOF ⟶ modest LDL-C ↓ (~13%) but ↓ C20:0 Cer (~20%) → ↓ CAD risk.
Clinical	Plasma (MI patients)	Systemic	MI → ↑ plasma C20:0 Cer (part of 12-ceramide prognostic signature) ⟶ predicted 12-month MACE (death, MI, stroke)
In vivo	Aortic tissue (human CABG biopsies)		CABG patients with recent MI ⟶ ↑ plasma C20:0 Cer, but not in aortic wall.
			Rat MI model ⟶ ↑ myocardial C20:0 Cer + ↑ expression of CerS enzymes → source of circulating ceramides.
[122]	Myocardium (rat LAD ligation)		Inferred mechanism: ischemia/reperfusion → Cer ↑ → Mitochondrial stress, apoptosis, inflammation → adverse remodeling + recurrent events.

Cer, ceramide; CerS, ceramide synthase; FXR, farnesoid X receptor; SPT, serine palmitoyltransferase; MMP, matrix metalloproteinase; ECM, extracellular matrix; COL1, collagen type I; FN1, fibronectin; AAD, aortic aneurysm and dissection; MI, myocardial infarction; LV, left ventricle; aCDase, acid ceramidase; S1P, sphingosine-1-phosphate; CAD, coronary artery disease; LDL-C, low-density lipoprotein cholesterol; CV, cardiovascular; MACE, major adverse cardiovascular events; VSMC, vascular smooth muscle cell; RE, endoplasmic reticulum; Mitoc, mitochondria; ↑: increase; ↓: decrease.

**Table 7:** Summary of C22:0 Ceramide Mechanisms Across Experimental and Clinical Context.

Scheme	Biological Matrix	Organelle Involved	Observed Pathophysiological Mechanism
In vivo	CerS2^+/−^ mice (chow vs. HFD)	RE + Mitoc	CerS2 haploinsufficiency → ↓ C22:0 Cer (and other VLCCs) → compensatory ↑ C16:0 Cer (via CerS5/6) ⟶ Mitoc dysfunction (↓ β-oxidation, ↓ ATP, impaired ETC activity) ⟶ lipid accumulation (↑ TAG, ↑ acylcarnitines) ⟶ hepatocyte apoptosis + macrophage infiltration ⟶ steatohepatitis + insulin resistance
In vitro [131]	Primary hepatocytes from CerS2^+/−^ mice		Myriocin ⟶ blocks de novo Cer synthesis ⟶ reverses phenotype CerS6 upregulation ⟶ mimics phenotype (↑ C16:0 ↓ C22:0/24:0 Cer) → impaired insulin signaling (↓ Akt-PKB) + ↑ steatosis.
In vitro	Hepatocytes HEK29 and J774A.1 (mice) (cells treated with ASOs)	RE + Mitoc	ASO silencing of CerS2 → ↓ C22:0 Cer (and ↓ C24:0/24:1 Cer) → compensatory ↑ LCCs (C16:0, C18:0 Cer) ⟶ altered sphingolipid profile in hepatocytes ⟶ modulation of plasma ceramides predictive of CV death
			Inferred mechanism: CerS2↓ → VLCCs (C22:0/24:0 Cer) ↓ → LCCs ↑ → pro-apoptotic/inflammatory signaling ↑ (via Cer16:0 Cer) → systemic ceramide imbalance → ↑ cardiometabolic risk.
In vivo [158]	Plasma (mice treated with GalNAc)		GalNAc-ASO ⟶ hepatocyte-specific delivery → potent, sustained ↓ CerS2 activity without hepatotoxicity → plasma ceramide signature shifted (↓ protective VLCCs, ↑ risk-associated LCCs)
Clinical	Plasma T2DM patients, responders vs. non-responders to metformin	Systemic	Inferred mechanism: ↑ *P.* vulgatus → bile acid deconjugation (↓ taurine-BAs) → intestinal FXR ↑ → ceramide synthase ↑ → C22:0 Cer ↑ (plasma + hepatocytes) ⟶ C22:0 Cer binds MFF → Mitoc fragmentation → OXPHOS ↓ → ATP ↓ ⟶ impaired β-oxidation + glucose utilization ↓ ⟶ hepatic insulin resistance + ↓ thermogenesis ⟶ metformin efficacy lost (MMF).
In vitro	HepG2 hepatocytes	RE + Mitoc	Exogenous C22:0 Cer in HepG2 ⟶ directly triggers MITOC fragmentation + ↓ OCR (Seahorse)
In vivo [159]	HFD-fed mice colonized with *P. vulgatus*		Interventions: cefaclor (↓ *P. vulgatus*) or adenosyl-cobalamin (VB12, ↑ MITOC function) ⟶ rescue metformin response.
In vivo	Hepatocytes (CRISPR knock-in mice harboring rs267738/E115A mutation in *Cers2*)	RE + Mitoc	*CerS2* SNP (E115A) → ↓ CerS2 activity (~42% in liver) → ↓ synthesis of VLCCs (C22:0, C24:0 Cer) → compensatory ↑ LCCs (C16:0 Cer) ⟶ Mitoc stress + impaired β-oxidation + ↓ insulin signaling (Akt) ⟶ hepatic steatosis + glucose intolerance (in HFD-fed mice).
	Adipose tissue (mice)		Subcutaneous white adipose tissue: ↑ C16:0 Cer accumulation
Clinical [160]	Serum (CAD cohort)	Systemic	Humans (CAD): SNP carriers showed no significant ↓ in serum VLCCs (C22:0/24:0 Cer) nor changes in CERT1 risk score, but genetic predisposition links to impaired glucose homeostasis and renal function
Clinical [146]	Plasma	RE + Mitoc	↑ C22:0 Cer plasma levels showed sex- and age-specific associations with cardiorespiratory (CR) fitness: In women < 54 y → inverse association with Wattmax/kg (↓ exercise capacity). In men ≥ 54 y → positive association with Wattmax/kg (↑ exercise capacity).
			Inferred mechanism: C22:0 Cer (VLCCs) generally linked to protective/neutral roles vs. LCCs (C16:0 Cer), but context dependent. Suggests that C22:0 Cer may modulate energy metabolism and CR fitness differently by sex/age, possibly via mitochondrial efficiency and lipid handling. Overall, ratios (C24:0/C16:0, C22:0/C16:0 Cer) were more robust predictors of cardiorespiratory fitness than single ceramides.

Cer, ceramide; CerS, ceramide synthase; VLCCs, very-long-chain ceramide; LCC, long-chain ceramide; RE, endoplasmic reticulum; Mitoc, mitochondria; ETC, electron transport chain; TAG, triacylglycerol; MFF, mitochondrial fission factor; Drp1, dynamin-related protein 1; OXPHOS, oxidative phosphorylation; OCR, oxygen consumption rate; FXR, farnesoid X receptor; T2DM, type 2 diabetes mellitus; CAD, coronary artery disease; CV, cardiovascular; CR, cardiorespiratory; HFD, high-fat diet; ASO, antisense oligonucleotide; SNP, single-nucleotide polymorphism; ↑: increase; ↓: decrease.

**Table 8:** Summary of C24:0 Ceramide Mechanisms Across Experimental and Clinical Context.

Study Type (Reference)	Biological Matrix	Organelle Involved	Observed Pathophysiological Mechanism
In vitro [163]	Cardiomyocytes (human siRNA knockdown of CERS2 vs. CERS5/6 ± PMA hypertrophy stimulus	RE + Mitoc	*CerS2* KD → ↓ C24:0 Cer (and other VLCCs) → loss of protective VLCCs pool → compensatory ↑ LCCs (C16:0 Cer) ⟶ altered transcriptomic response to hypertrophic stimulus (PMA) CerS5/6 KD (↓ C16:0 Cer) ⟶ favorable hypertrophy response (attenuated maladaptive signaling)
			Inferred mechanism: ↓ C24:0 Cer → dysregulation of lipid/cholesterol metabolism, ECM remodeling, immune/inflammatory signaling, SREBP pathways ⟶ exacerbated hypertrophic phenotype (↑ BNP, ↑ cell size, maladaptive remodeling)
In vitro [164]	Colon cancer cells (HCT-116) upregulation and co-expression of CerS2, CerS4, CerS6; ± ELOVL1 knockdown	RE + Mitoc	CerS2 alone upregulation→ no major ↑ in C24:0 Cer unless VLCCs-CoA substrates supplied CerS2 + CerS4/6 co-expression → ↑↑ C24:0/C24:1-Cer
			ELOVL1 knockdown → ↓ C24:0 Cer synthesis (substrate limitation)
			Inferred mechanism: C24:0 Cer ↑ → equilibrium LCCs/VLCCs restored → ↓ apoptosis, ↓ cell cycle arrest, ↑ survival/colony formation In contrast, LCCs ↑ (C16:0, C18:0 Cer) → apoptosis ↑ + proliferation ↓. Balance shift toward VLCCs (C24:0 Cer) ⟶ counteracts pro-apoptotic/proliferative effects of LCCs (C16:0, C18:0 Cer)
Clinical	Plasma (obese vs. obese-T2DM subjects)	Systemic	C24:0 Cer ↑ (plasma, adipose, liver) → impairs adipocyte differentiation & lipid storage ⟶ hypertrophic WAT, ↓ remodeling ⟶ ectopic lipid spillover.
In vitro	Adipocytes (*3T3-L1*, WAT) Hepatocytes (*HUH7*, mouse)	RE + Mitoc	Adipose storage failure + hepatic steatosis → glucose imbalance, insulin resistance, T2DM phenotype. In hepatocytes: C24:0 Cer → Mitoc dysfunction (OCR ↓, ATP ↓, ROS ↑, Ca^2+^ overload) + ER stress activation ⟶ steatosis + insulin resistance.
In vivo [165]	Offspring of HFD-diet rats; chronic C24:0 administration in *C57BL/6* mice		Fetal programming (HFD diet) → early ↑ plasma C24:0 Cer in offspring ⟶ predisposition to metabolic disease.
Clinical [166]	Plasma and serum (patients with SLE ± lupus nephritis)	Systemic	LN → ↑ C24:1 Cer and C24:1 dhCer in plasma/serum ⟶ correlated with proteinuria, ↓ eGFR, ↑ creatinine
			Inferred mechanism: C24:1 Cer accumulation → imbalance of LCCs/VLCCs ceramides → enhanced inflammatory signaling (inverse correlation with complement C3/C4) → renal injury progression
			ROC analysis: serum C24:1 Cer showed AUC > 0.9 to differentiate LN from SLE without renal damage ⟶ strong, stable biomarker (not influenced by glucocorticoid therapy)
Clinical [44,145,151]	Serum and Plasma (humans: T1DM, CAD/ACS patients)	Systemic	C24:0 Cer often protective—higher levels linked to ↓ all-cause mortality (T1DM), but paradoxically ↑ CVD risk in PREDIMED when isolated
			C24:1 Cer consistently pathogenic—↑ in CAD/ACS and LN, associated with CV death
			Ratios (C16:0/C24:0, C18:0/C24:0, C24:1/C24:0 Cer): strongest predictors of CV events and mortality (HR/OR up to 10) Inferred mechanism: imbalance ↑ LCCs/↓ VLCCs → ↑ endothelial dysfunction, inflammation, apoptosis, plaque instability, insulin resistance. Mediterranean diet attenuates risk despite high ceramides.

Cer, ceramide; CerS, ceramide synthase; VLCCs, very-long-chain ceramides; LCCs, long-chain ceramides; RE, endoplasmic reticulum; Mitoc, mitochondria; ETC, electron transport chain; TAG, triacylglycerol; ECM, extracellular matrix; SREBP, sterol regulatory element-binding protein; JNK, c-Jun N-terminal kinase; OCR, oxygen consumption rate; ROS, reactive oxygen species; T1DM, type 1 diabetes mellitus; T2DM, type 2 diabetes mellitus; CAD, coronary artery disease; ACS, acute coronary syndrome; CV, cardiovascular; MS, metabolic syndrome; SLE, systemic lupus erythematosus; LN, lupus nephritis; eGFR, estimated glomerular filtration rate; ↑: increase; ↓: decrease.

**Table 9:** Pharmacological and non-pharmacological strategies modulating ceramide metabolism and associated physiological effects.

Intervention	Agent/Reference	Molecular Mechanism	Ceramide Species Affected	Physiological/Therapeutic Effect	Application/Experimental Model
Pharmacological	Statins [170,173,174,175]	Inhibit HMG-CoA reductase→↓ ER cholesterol→activation of SREBP-2. Indirect modulation of phospholipid and sphingolipid biosynthetic enzymes.	C16:0 Cer, C24:0 Cer, C24:1 Cer; HexCer 16:0; SM 18:1/16:0; very-long-chain ceramides (C22:0, C24:0, C24:1).	LDL-C reduction (−38% to −49%) and ceramide decrease (−23% to −52%). Partial normalization of lipid signatures; strong correlation with apoC-III.	Indirect reduction of residual cardiovascular risk.
	Ezetimibe [155,175,176]	Selective NPC1L1 inhibitor→reduces intestinal cholesterol and SM absorption→apoM-associated S1P production.	Neutral or modest increase in C18:0 Cer and C20:0 Cer. No reduction in circulating ceramides.	LDL-C reduction (~21%) but no additional ceramide benefit. Ceramide ratios are associated with cardiovascular risk.	Clinical cohorts for cardiovascular risk assessment.
	PCSK9 inhibitors [155,180,181]	PCSK9→LDLR and CD36 lysosomal degradation. Inhibition (monoclonal antibodies, siRNA) increases surface LDLR/CD36→enhanced lipid uptake.	PCSK9 deficiency linked to decreased ceramides and cerebrosides (~25%). Evidence emerging for direct sphingolipid modulation.	Potent LDL-C reduction with concomitant ceramide decrease; improved cardiovascular risk prediction beyond LDL-C.	Therapies in dyslipidemia; clinical studies of cardiovascular risk, PCSK9 and CD36 regulation.
	Fumonisin B1 (competitive inhibitor of CerS) [182]	Structurally mimics sphingoid bases → binds CerS active site ⟶ blocks acylation with acyl-CoA.	↓ dhCer, Cer, complex sphingolipids; ↑ dhSph, Sph, phosphorylated forms.	Experimental Tool for studying sphingolipid biosynthesis and Cer-mediated signaling.	Model to explore therapies of diseases caused by sphingolipid accumulation; limited by toxicity.
	Myriocin (SPT inhibitor) [183]	Blocks SPT → ↓ 3-KDS, dhSph ⟶ limits de novo dhCer/Cer synthesis.	↓ Total dhCer and Cer (de novo pathway).	Improves glucose homeostasis and insulin sensitivity (↑ Akt/PKB phosphorylation, ↓ FFAs, TAG).	Preclinical candidate for obesity, diabetes, and CVD.
	Fenretinide (Des1 inhibitor) [184]	Synthetic retinoid; RAR-dependent signaling; directly inhibits Des1 ⟶ prevents dhCer→Cer conversion.	↓ C18:0, C20:0, C22:0; ↑ dhCer species.	Improves glucose tolerance; ↓ hyperglycemia; ↓ hepatic Cer; ↑ insulin sensitivity.	Preclinical and early clinical candidate for obesity, T2DM, MASLD.
	Ceranib-1/2 (non-lipid CDase inhibitors) [185]	Prevents Cer→Sph hydrolysis ⟶ ↓ S1P formation.	↑ Total Cer (Ceranib-2 > 100% vs. control).	Inhibits tumor growth, promotes apoptosis, induces cell-cycle arrest.	Cancer and creation of useful targets for metabolic/cardiovascular disease research: selective modulators to reduce harmful ceramides while preserving signaling.
	Synthetic ceramide analogues (315 and 403) [186].	Activate CerS; suppress SphK1/2, glucosylceramide synthase ⟶ shift Cer/S1P balance toward apoptosis.	↑ C16:0, C18:0, C18:1, C20:0 (2.5–4× total Cer).	Induce caspase-dependent apoptosis; G1/G2-M arrest; ↓ cyclin D1/CDK6, ↑ p21.	
	Antisense oligonucleotides (ASOs) [131].	Bind mRNA of ceramide-synthesis enzymes ⟶ degradation/block translation.	↓ C16:0 and pathological species; DES1-ASO ↑ dhCer, ↓ mature Cer.	↑ insulin sensitivity; ↓ hepatic steatosis, inflammation, and obesity.	Obese/insulin-resistant mice; potential for T2DM, MASLD, CVD.
	Fingolimod (FTY720) [187]	Sph analog; S1P receptor modulator; competitively inhibits CerS via dhSph	↓ LC and VLC Cer (isoform-dependent)	↓ pathological Cer; ↑ mitochondrial function and insulin sensitivity	Tested in HFD mice; tool for obesity, insulin resistance, fatty liver
	Nogo-A [188,189] (SPT inhibiton) (endogenous mechanism with translational potential)	Direct binding to SPTLC1 at the ER → negative regulation of serine palmitoyltransferase (SPT) activity → ↓ ceramide de novo biosynthesis.	↓ accumulation of LCCs (C16–C22); preservation of mitochondrial function and autophagy.	Protects cardiomyocytes from lipotoxicity, maladaptive hypertrophy, and contractile dysfunction; improves survival under pressure overload.	Potential therapeutic target to limit ceramide accrual in cardiomyocytes, preserving autophagy and mitochondrial function; translation relevance for preventing progression to heart failure under sustained hemodynamic stress.
Non- pharmacological	Dietary intervention [190]	Fruits, vegetables, fiber, PUFAs, polyphenols ⟶ ↓ palmitate intake, modulate CerS/SMase.	↓ C16:0, C18:0, C24:0, C24:1; ↑ C22–C24 ratio (protective)	↑ insulin sensitivity; ↓ steatosis/inflammation; neuroprotection	Prevention/treatment: MS, T2DM, MASLD, CVD and NDD.
	Exercise (prolonged/resistance) [191]	Modulates nSMase and de novo synthesis in skeletal muscle.	↑ C16 and C18:0 (adaptive)	Regulates glucose uptake, improves insulin sensitivity, muscle metabolism.	Prevention of insulin resistance and muscle dysfunction.

CerS: ceramide synthase; SPT: serine palmitoyltransferase; Des1: dihydroceramide desaturase 1; CDase: ceramidase; Sph: sphingosine; S1P: sphingosine-1-phosphate; dhCer: dihydroceramide; dhSph: dihydrosphingosine; FFA: free fatty acids; TAG: triglycerides; RAR: retinoic acid receptor; CVD: cardiovascular disease; T2DM: type 2 diabetes mellitus; MASLD: metabolic dysfunction-associated steatotic liver disease; NDD: neurodegenerative disease; HFD: high-fat diet; LCCs/VLCCs: long-/very-long-chain ceramides; Akt/PKB: protein kinase B; SMase: sphingomyelinase; ASO: antisense oligonucleotide.

## Data Availability

No new data were created or analyzed in this study.

## Abbreviations

The following abbreviations are used in this manuscript:

3-KDS	3-ketosphinganine
3-KDSR	3-ketosphinganine reductase
AA	Arachidonic acid
AAD	aortic aneurysm and dissection
ACDase	Acid ceramidase
ACS	Acute Coronary Syndrome
AFB1	Aflatoxin B1
Akt/PKB	Protein Kinase B.
AlkCDases	Alkaline ceramidases
AlkSMase	Alkaline sphingomyelinase
ASC	Apoptosis-associated speck-like protein
ASCVD	Atherosclerotic cardiovascular disease
ASO	Antisense oligonucleotide
Atg7	Autophagy-related gene 7
ATP	Adenosine triphosphate
AUC	Area under the curve
BAPN	β-aminopropionitrile
BAT	Brown Adipose Tissue
Bax/Bak	Pro-apoptotic Bcl-2 proteins
BECN1	Beclin-1
BMDM	Bone Marrow-Derived Macrophages
BMI	Body mass index
BNP	Brain Natriuretic Peptide
BP	Blood Pressure
CABG	Coronary Artery Bypass Grafting
CAD	Coronary artery disease
CDase	Ceramidase
Cer	Ceramide
CerS	Ceramide synthase
CERT	Ceramide transfer protein
CERT1, CERT2	Ceramide risk scores 1 and 2
CHOP	C/EBP Homologous Protein
CI	Confidence interval
CoA	Coenzyme A
COL1	Collagen Type I
CV	Cardiovascular
CVD	Cardiovascular disease
DAMP	Damage-associated molecular pattern
DES	Dihydroceramide desaturase
DhCer	Dihydroceramide
DhSph	Dihydrosphingosine (sphinganine)
DISC	Death-inducing signaling complex
Drp1	Dynamin-related protein 1
ECM	Extracellular Matrix
EGFR	Estimated Glomerular Filtration Rate
EIF2α	Eukaryotic Initiation Factor 2 alpha
ELOVL1	Elongation of very long chain fatty acids protein 1
ELISA	Enzyme-Linked Immunosorbent Assay
ENOS	Endothelial nitric oxide synthase
ER	Endoplasmic reticulum
ETC	Electron Transport Chain
EVOO	Extra Virgin Olive Oil
EVs	Extracellular Vesicles
F1B	Fumonisin B1
FA	Fatty Acid.
FFA	Free fatty acid
FN1	Fibronectin 1
FXR	Farnesoid X Receptor
GalNAc	N-acetylgalactosamine conjugate
GCS	Global circumferential strain
GFP	Green fluorescent protein
GLUT4	Glucose transporter type 4
HF	Heart failure
HFD	High-fat diet
HMG-CoA	3-Hydroxy-3-Methylglutaryl-CoA
HOMA-IR	Homeostatic model assessment of insulin resistance
HR	Hazard ratio
HRmax	Maximum heart rate
HSL	Hormone-sensitive lipase
HUH7	Human Hepatoma Cell Line
HUVEC	Human umbilical vein endothelial cells
IL	Interleukin
IL-1β	Interleukin 1-beta
IMF	Intramyofibrillar
IR	Insulin Resistance
L-aSMase	lysosomal acid sphingomyelinase
LAD	Left Anterior Descending artery ligation
LAEF	left atrial emptying fraction
LASS	Longevity Assurance genes
LAVes	left atrial end-systolic volume
LC3B	Microtubule-associated protein 1A/1B-light chain 3B
LCCs	Long Chain Ceramides
LDL-C	low-density lipoprotein cholesterol
LDLR	Low-Density Lipoprotein Receptor
LN	lupus nephritis
LOF	Loss of Function
LPP	long periodicity phase
LV	Left Ventricle
LVEF	left ventricular ejection fraction
MA	Myristic acid
MACE	Major adverse cardiovascular events
MALDI-IMS-FTICR	Matrix-Assisted Laser Desorption/Ionization—Imaging Mass Spectrometry—Fourier Transform Ion Cyclotron Resonance
MAPKs	Mitogen-activated protein kinases
MASLD	Metabolic dysfunction-associated liver disease
MeSH	Medical Subject Headings
MFF	Mitochondrial fission factor
MI	Myocardial infarction
Mitoc	Mitochondria
MMF	Metformin Metabolic Failure
MMP	Matrix metalloproteinases
ModRNA	Modified messenger RNA
MOMP	Mitochondrial outer membrane permeabilization
MR	Mineralocorticoid receptor
MRM	Multiple Reaction Monitoring
MRNA	Messenger RNA
MS	Metabolic syndrome
NCDase	Neutral ceramidase
NFκB	Nuclear factor kappa-light-chain-enhancer of activated B cells
NLRP3	NOD-, LRR- and pyrin domain-containing protein 3
NPLC	Normal Phase Liquid Chromatography
NPC1L1	Niemann-Pick C1-Like 1
NO	Nitric oxide
NSMase	Neutral sphingomyelinase
OCR	Oxygen consumption rate
OR	Odds ratio
OXPHOS	Oxidative phosphorylation
PA	Palmitic acid
Pal-CoA	Palmitoyl-CoA
PCSK9	Proprotein Convertase Subtilisin/Kexin Type 9
PDMP	D-threo-1-Phenyl-2-decanoylamino-3-morpholino-1-propanol (glucosylceramide synthase inhibitor)
PERK	Protein kinase RNA-like endoplasmic reticulum kinase
PG	Prostaglandins
PKCζ	Protein kinase C zeta isoform
PLA_2_	Phospholipase A_2_
PM	Plasma Membrane
PMA	Phorbol 12-myristate 13-acetate
PP2A	Protein phosphatase 2A
PUFA	Polyunsaturated fatty acid
ROC	Receiver Operating Characteristic
ROS	Reactive oxygen species
RPLC	Reverse Phase Liquid Chromatography
S1P	Sphingosine-1-phosphate
SaSMase	Secretory acid sphingomyelinase
SC	Stratum corneum
ScAT	Subcutaneous Adipose Tissue
SCD	Sudden cardiac death
SCD1	Stearoyl-CoA Desaturase 1
Ser	Serine
SGMS1	Sphingomyelin synthase 1
ShRNA	Short Hairpin RNA
SiRNA	Small Interfering RNA
SLE	Systemic lupus erythematosus
SM	Sphingomyelin
SMase	Sphingomyelinase
SNP	Single Nucleotide Polymorphism.
Sph	Sphingosine
SphK	Sphingosine kinase
SPP1	S1P phosphatase 1
SPR	Surface plasmon resonance
SPT	Serine palmitoyltransferase
SPT2	Serine Palmitoyltransferase 2
SPTLC2/3	Serine Palmitoyltransferase Long Chain Base Subunits 2/3
SREBP	Sterol Regulatory Element-Binding Protein
SS	Subsarcolemmal
STEMI	ST-segment elevation myocardial infarction
SWAT	Subcutaneous White Adipose Tissue
T1DM	Type 1 Diabetes Mellitus
T2DM	Type 2 diabetes mellitus
TAA	Thoracic aortic aneurysm
TAC	Transverse aortic constriction
TAD	Thoracic aortic dissection
TAG	Triacylglycerol
tBid	Truncated Bid
TCA	Tricarballylic acid
TAG	Triglycerides
ThAT	Thoracic Adipose Tissue
TLR4	Toll-like receptor 4
TNF-α	Tumor necrosis factor alpha
UDCA	Ursodeoxycholic Acid
ULCCs	Ultralong-chain ceramide
UV	Ultraviolet
VAD	Ventricular assistance device
VB12	Vitamin B12 (adenosylcobalamin)
VDAC2	Voltage-dependent anion channel 2
VLCCs	Very Long Chain Ceramides
VLDL	Very Low Density Lipoprotein
VSMCs	Vascular Smooth Muscle Cells
WAT	White Adipose Tissue
